# Tiny pollen grains: first evidence of Saururaceae from the Late Cretaceous of western North America

**DOI:** 10.7717/peerj.3434

**Published:** 2017-06-13

**Authors:** Friðgeir Grímsson, Guido W. Grimm, Reinhard Zetter

**Affiliations:** 1 Department of Palaeontology, University of Vienna, Vienna, Austria; 2 Unaffiliated, Orléans, France

**Keywords:** Angiosperm evolution, Conservative traits, Piperales, Molecular dating, Magnoliids, Paleophytogeography, *Saururus*

## Abstract

**Background:**

The Saururaceae, a very small family of Piperales comprising only six species in four genera, have a relatively scanty fossil record outside of Europe. The phylogenetic relationships of the four genera to each other are resolved, with the type genus *Saururus* occurring in both eastern North America and East Asia. No extant species occurs in western Eurasia. The most exceptional find so far has been an inflorescence with *in-situ* pollen, *Saururus tuckerae* S.Y.Sm. & Stockey from Eocene of North America with strong affinities to extant species of *Saururus.* Recent dated trees suggest, however, an Eocene or younger crown age for the family.

**Methods:**

Dispersed fossil pollen grains from the Campanian (82–81 Ma) of North America are compared to dispersed pollen grains from the Eocene strata containing *S. tuckerae,* the Miocene of Europe, and extant members of the family using combined LM and SEM imaging.

**Results:**

The unambiguous fossil record of the Saururaceae is pushed back into the Campanian (82–81 Ma). Comparison with re-investigated pollen from the Eocene of North America, the Miocene of Europe, and modern species of the family shows that pollen morphology in Saururaceae is highly conservative, and remained largely unchanged for the last 80 million years.

**Discussion:**

Campanian pollen of Saururaceae precludes young (Eocene or younger) estimates for the Saururaceae root and crown age, but is in-line with maximum age scenarios. *Saururus-*type pollen appear to represent the primitive pollen morphology of the family. Often overlooked because of its small size, dispersed Saururaceae pollen may provide a unique opportunity to map the geographic history of a small but old group of Piperales, and should be searched for in Paleogene and Cretaceous sediment samples.

## Introduction

Smith & Stockey (2007a) described inflorescences and flowers with *in-situ* pollen from the Eocene of North America that they assigned then to the modern genus *Saururus* (*S. tuckerae* S.Y.Sm. & Stockey). Saururaceae are a very small magnoliid family included in the Piperales (APG III, 2009), with six currently accepted species in four genera. In addition to *Saururus cernuus* L. and *S. chinensis* (Lour.) Baill., these are: *Anemopsis californica* Hook. & Arn., *Gymnotheca chinensis* Decne., *Gymnotheca involucrata* Pei, and *Houttuynia cordata* Thunb. An interesting pattern is the modern disjunct distribution of both of the two mutually monophyletic lineages in the Saururaceae (*Anemopsis* + *Houttuynia* vs. *Gymnotheca* + *Saururus*; Massoni, Forest & Sauquet, 2014) in North America and South/East Asia, suggesting that the family probably had a much wider distribution in the past (Table 1). The fossil record of Saururaceae is scanty (Table 2). Most of the fossils are fruits/seeds from the Eocene to Pliocene of western Eurasia and have been assigned to *Saururus* (*S. bilobatus* [Nikitin] Mai). In addition, Mai (1999) described fruits/seeds from the lower Miocene of Germany as *Houttuynia bavarica* Mai. The oldest fossil record so far is fossil wood from the Upper Cretaceous (no detailed stratigraphic information available) of Hokkaido described as *Saururopsis niponensis* Stopes & Fujii (1910, p. 58ff); the authors discuss carefully the affinity of the fossil and suggest that it could represent an ancestral member of the Saururaceae combining wood features typical for either *Saururus* or *Houttuynia.* The Eocene *Saururus tuckerae* (Smith & Stockey, 2007a) is so far the only fossil reported from North America. Though scanty, the fossil record confirms that Saururaceae were widespread by the Paleogene. The fossil record is also in line with the latest molecular dating estimates of a magnoliid dataset. According to the dating analyses of Massoni, Couvreur & Sauquet (2015b), the divergence between the two clades of the Saururaceae (*Anemopsis* +* Houttuynia* vs. *Gymnotheca* + *Saururus*) was established at the latest by the Eocene (>45 Ma), and the modern genera (and disjunctions) by the late Miocene (>10 Ma; Table 3). Two nodes in the phylogenetic neighbourhood of the Saururaceae were constrained using fossil age priors: the *Saururus* (≥44.3 Ma; ‘safe’ minimum constraint with reference to *S. tuckerae*) and Winteraceae root ages (=Canellales crown age; ≥ 126 Ma; Massoni, Doyle & Sauquet, 2015). Here, we document fossil pollen from the middle Upper Cretaceous Eagle Formation (Fm) of Wyoming, western North America, that is very similar to those of extant *Saururus* and nearly identical to that of pollen recovered *in situ* from *Saururus tuckerae* from the Eocene of British Columbia. Our findings are discussed in the context of newly documented dispersed *Saururus* pollen from the Eocene of British Columbia and Miocene of Central Europe (Austria), and the recent dating estimates for the family.

**Table 1:** Modern and past distribution of Saururaceae genera.

Time period	North America	Western Eurasia	East Asia
Recent	*Saururus, Anemopsis*	None	*Gymnotheca, Saururus, Houttuynia*
Neogene	None	*Saururus* (pollen, fruit/seed), *Houttuynia* (fruit/seed)	None
Paleogene	*Saururus* (inflorescence with *in-situ* pollen; and dispersed pollen)	*Saururus* (fruit/seed)	*Saururus* (fruit/seed)
Upper Cretaceous	*Saururus*-type pollen	None	*Saururopsis* (wood)

**Table 2:** Fossil record of Saururaceae.

Taxon	Organ	Period (epoch)	Age in Ma	State/region, country	Reference
**North America**					
*Saururus aquilae* sp. nov.	Pollen	Late Cretaceous (Campanian)	82–81	Wyoming, United States	This study
*Saururus tuckerae*	Inflorescence, flowers, pollen	Middle Eocene	∼48	British Columbia, Canada	Smith & Stockey (2007a), This study
**Western Eurasia**					
*Saururus bilobatus*	Fruits/seeds	Late Eocene to Pliocene	∼40–2.5	Germany	Reid & Reid (1915), Mai (1965), Mai (1967), Mai & Walther (1978), Mai & Walther (1985), Mai (1995), Mai (1999)
*Saururus bilobatus* (incl. *Helitropium* sp. and *Carpolithus* sp.)	Fruits/seeds	Miocene	∼23–5	Poland	Raniecka-Bobrowska (1959), Łańcucka-Środoniowa (1979), Stuchlik et al. (1990), Lesiak (1994)
*Saururus bilobatus*	Fruits/seeds	Middle Miocene (Langhian)	∼16–14	Denmark	Friis (1985)
*Saururus stoobensis* sp. nov.	Pollen	Late Miocene (Tortonian to Messinian)	∼12–6	Austria	Ferguson, Zetter & Paudayal (2007) as “*Saururipollis* sp.” (nomen nudum); formalized in this study
*Houttuynia bavarica*	Fruits/seeds	Early Miocene	∼23–16	Germany	Mai (1999)
**East Asia**					
*Saururopsis niponensis*	Wood	Late Cretaceous	>66	Hokkaido, Japan	Stopes & Fujii (1910)
*Saururus bilobatus* (as *Carpolithus bilobatus*)	Fruits/seeds	Oligocene	∼34–23	Western Siberia, Russia	Dorofeyev (1963), Nikitin (1965)

**Table 3:** Divergence age estimates for the Saururaceae subtree according to minimum and maximum age scenarios (angiosperm root fixed to max. 130 or 200 Ma; Massoni, Couvreur & Sauquet, 2015a).

Node	Angiosperm root fixed to	
	Max. 130 Ma	Max. 200 Ma
Saururaceae root	97.0–60.4 (median: 78.3)	117.3–80.3 (median: 99.8)
Saururaceae crown	75.3–46.7 (median: 58.9)	80.8–48.6 (median: 62.8)
MRCA of *Gymnotheca*+*Saururus*	62.1–44.3 (median: 49.4)	65.6–44.3 (median: 50.6)
MRCA of *Anemopsis*+*Houttuynia*	64.5–10.3 (median: 37.1)	72.5–26.8 (median: 49.5)

**Notes.**

Abbreviations MRCA most recent common ancestor Ma Million years ago

## Material & Methods

### Palaeopalynological samples

The sedimentary rock samples containing the dispersed fossil *Saururus* pollen grains presented in this study originate from three different localities:

(1) the Elk basin, Wyoming, north-western United States (44°59′N/108°52′W); the sediment sample comes from the Campanian Eagle Fm and was provided by the late Leo Hickey (1940–2013). For detailed chronometric (absolute dating of the overlying benthonite; Hicks, 1993) and stratigraphic information and palaeobotanical background of this locality see Hicks (1993), Van Boskirk (1998), Manchester, Grímsson & Zetter (2015), and Grímsson et al. (2016a).
(2) an outcrop of the Princeton Chert beds, Similkameen River, British Columbia, Canada (49°22′N, 120°32′W). The Princeton Chert beds are part of the middle Eocene Allenby Fm and comprise at least 49 rhythmically bedded cherts, interbedded by carbonaceous layers (e.g., Read, 2000; Smith & Stockey, 2007a; Mustoe, 2011). The sample originates from chert-bed 43 (uppermost quarter of the Princeton Chert unit) and was provided by Ruth Stockey. Overlaying and underlying beds have been chronometrically dated. According to Moss, Greenwood & Archibald (2005, fig. 2) an age of c. 48 Ma can be assumed for this part of the formation.
(3) An open cast clay pit, Stoob-Warasdorf-Forest, Burgenland, Austria. No chronometric dates are available; bio- and lithostratigraphy indicate a late Miocene age (Klaus, 1982).

### Sample preparation and the single grain method

The sediment samples were processed and pollen grains extracted according to the protocol outlined in Grímsson, Denk & Zetter (2008). The fossil Saururaceae pollen grains were investigated both by light microscopy (LM) and scanning electron microscopy (SEM) using the single grain method described in Zetter (1989).

### Pollen descriptions and comparison to extant material

The description of the fossil pollen grains includes diagnostic features observed both in LM and SEM. Some grains were deliberately broken to expose the pollen wall to measure the exine and nexine thickness using SEM. TEM measurements for *Saururus tuckerae* are based on Smith & Stockey (2007a, fig. 29) and Smith & Stockey (2007b, fig. 12B). Pollen terminology follows Punt et al. (2007) and Hesse et al. (2009). The fossil pollen grains were compared to all previously published Saururaceae pollen that have been documented using LM and SEM (Xi, 1980; Takahashi, 1986; Pontieri & Sage, 1999; Sampson, 2000; Furness, Rudall & Sampson, 2002; Smith & Stockey, 2007a; Smith & Stockey, 2007b; Lu et al., 2015). Additional material (Table S1) from the herbarium of the University of Vienna (WU) was used for a more detailed comparison (pollen figured in File S1).

### Preparation of extant material

A single or a few anthers from each sample were placed into drops of acetolysis liquid (nine to one mix of 99% acetic anhydride and 95–97% sulphuric acid) on microscopic glass slides to soften up the anthers, release the pollen grains from anthers, dissolve extra organic material on pollen grain surfaces, rehydrate pollen grains and release their cell contents, and finally, to stain the grains for LM photography. The slides were heated over a candle flame to speed up the process. Pollen grains were then transferred into fresh drops of glycerine and photographed under LM and then transferred to SEM stubs using a micromanipulator and washed with drops of absolute ethanol. Stubs were sputter-coated with gold and the pollen grains photographed under a JEOL 6400 SEM.

### Conservation of fossil and extant pollen material

SEM stubs produced for this study are stored in the collection of the Department of Palaeontology, University of Vienna, Austria, under accession numbers IPUW 7513/101–130.

## Systematic Palaeobotany

**Figure 1:**
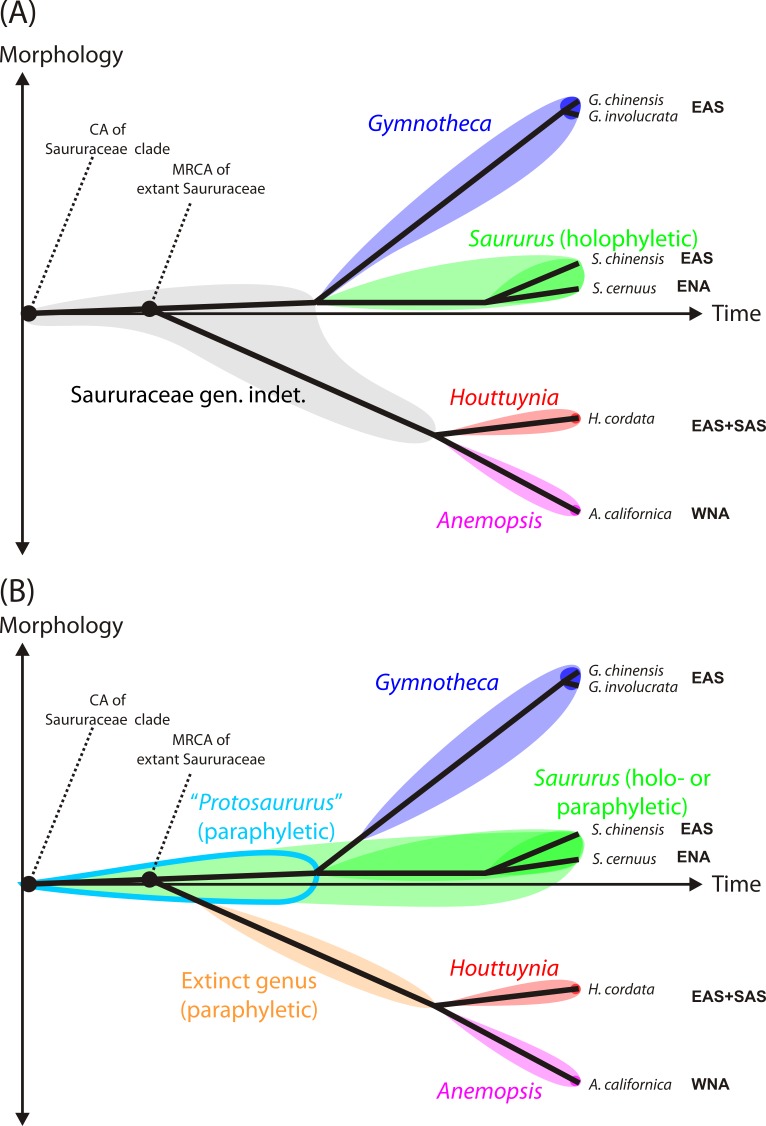
Practical shortcoming of cladistic classification for naming fossil and extant members of phylogenetic lineages (clades) using binominals. Shown are schematic phenograms using the current systematic-phylogenetic framework for extant taxa of the family (Massoni, Couvreur & Sauquet, 2015b). (A) Cladistic classification of Saururaceae accepting only holophyletic (Ashlock, 1971), i.e., inclusively monophyletic groups: *All* organisms descending from a certain common ancestor are addressed by the same genus name. All stem fossils must be named ‘Saururaceae gen. et spec. indet.’, unless there is conclusive evidence that they represent extinct sister lineages with no ancestor-descendant relationship to the extant genera (triggering the erection of a new genus) or belong to the stem or crown lineages of an extant genus. (B) Evolutionary classification, accepting groups with inclusive (holophyla) and exclusive (paraphyla) common origins, i.e., are monophyletic according to Haeckel (1866). All fossil taxa can be named using binominals, either by extending a today holophyletic genus to include ancestral members of Saururaceae with equally primitive morphology, which then becomes paraphyletic by definition (e.g., *Saururus*), or by introducing genera to collect stem fossils ancestral to more than a single, extant and holophyletic genus (e.g., the tentative *Protosaururus* to collect fossils with *Saururus*-like morphology that are older than the presumed split between *Saururus* and *Gymnotheca*-lineages). Such extinct genera are also paraphyletic by definition. Shading signifies the extent of each (potential) genus, dark shading the modern circumscription based on molecular data (i.e., descendants of the MRCA of all extant species of the genus). Abbreviations: CA, common ancestor; MRCA, most recent common ancestor; EAS, East Asia; ENA, Eastern North America; SAS, South Asia (Indian Peninsula); WNA, Western North America.

**Nomenclatural note**. We believe that a fossil name should reflect the biological affinity indicated by the morphology of the fossil. Taking together all evidence, our Cretaceous pollen grains either represent an ancestral lineage within the Saururaceae that shared the primitive pollen morphology of extant and Cenozoic *Saururus* (hypothesis 1 below) or an early member of the *Saururus*-lineage (hypothesis 2). A name best reflecting hypothesis 1 would be to erect a new genus named, e.g. “*Protosaururus*” (Fig. 1B). However, this is impractical. The genus diagnosis could only be based on the Cretaceous pollen grains, and would be non-exclusive regarding pollen of the actual *Saururus*-lineage. If the currently prevalent cladistic-phylogenetic nomenclature should be followed (Fig. 1A) that only accepts taxa that have a (putative) inclusive common origin, i.e., are ‘monophyletic’ in a strict sense (Hennig, 1950; Hennig & Schlee, 1978), termed also ‘holophyletic’ by Ashlock (1971), the Cretaceous fossils would need to be addressed as “Saururaceae gen. et sp. indet.” (hypothesis 1) or *Saururus* (hypothesis 2). For consistency, our and future *Saururus*-type pollen grains would need to be named based on the currently accepted divergence ages for the Saururaceae (Fig. 1). An alternative solution that serves the requirements of the Botanical Code for unambiguous diagnoses is to follow the concept of “evolutionary classification” (e.g., Mayr & Bock, 2002; Hörandl, 2006; Hörandl, 2007), which allows naming also ‘paraphyletic’ groups to avoid that groups of directly related organisms with a non-inclusive common origin and similar or identical morphology are addressed by different names (Fig. 1B). In this case, one does not need to decide which hypothesis (paraphyletic *Saururus* pollen vs. holophyletic *Saururus*) applies when naming the pollen; and all *Saururus*-type pollen can be addressed as *Saururus* spp.

**Figure 2:**
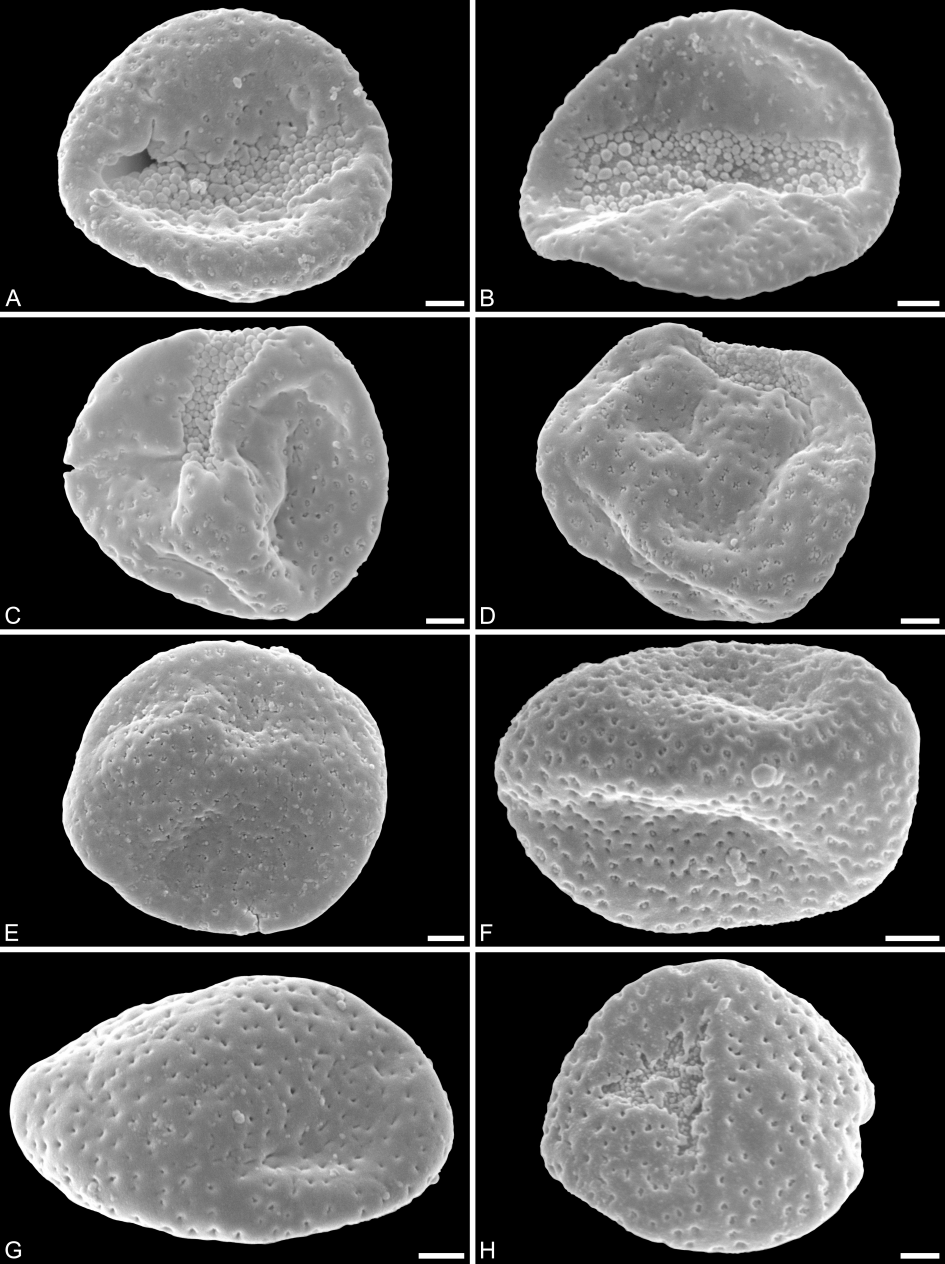
SEM micrographs of *Saururus aquilae* sp. nov. from the Upper Cretaceous (Campanian, 82–81 Ma) of Wyoming, western USA. (A) Holotype, IPUW 7513/101; pollen grain in distal polar view, showing sulcus, microechini densely packed. (B) Paratype, IPUW 7513/102; pollen grain in distal polar view, showing sulcus, microechini segregated. (C) Paratype, IPUW 7513/103; pollen grain in equatorial view, showing sulcus. (D) Paratype, IPUW 7513/104; pollen grain in equatorial view, showing sulcus. (E) Paratype, IPUW 7513/105; pollen grain in proximal polar view. (F) Paratype, IPUW 7513/106, pollen grain in proximal polar view. (G) Paratype, IPUW 7513/107, pollen grain in proximal polar view. (H) Paratype, IPUW 7513/108; pollen grain in proximal polar view, with eroded parts revealing the columellae. Scale bars: 1 μm.

**Figure 3:**
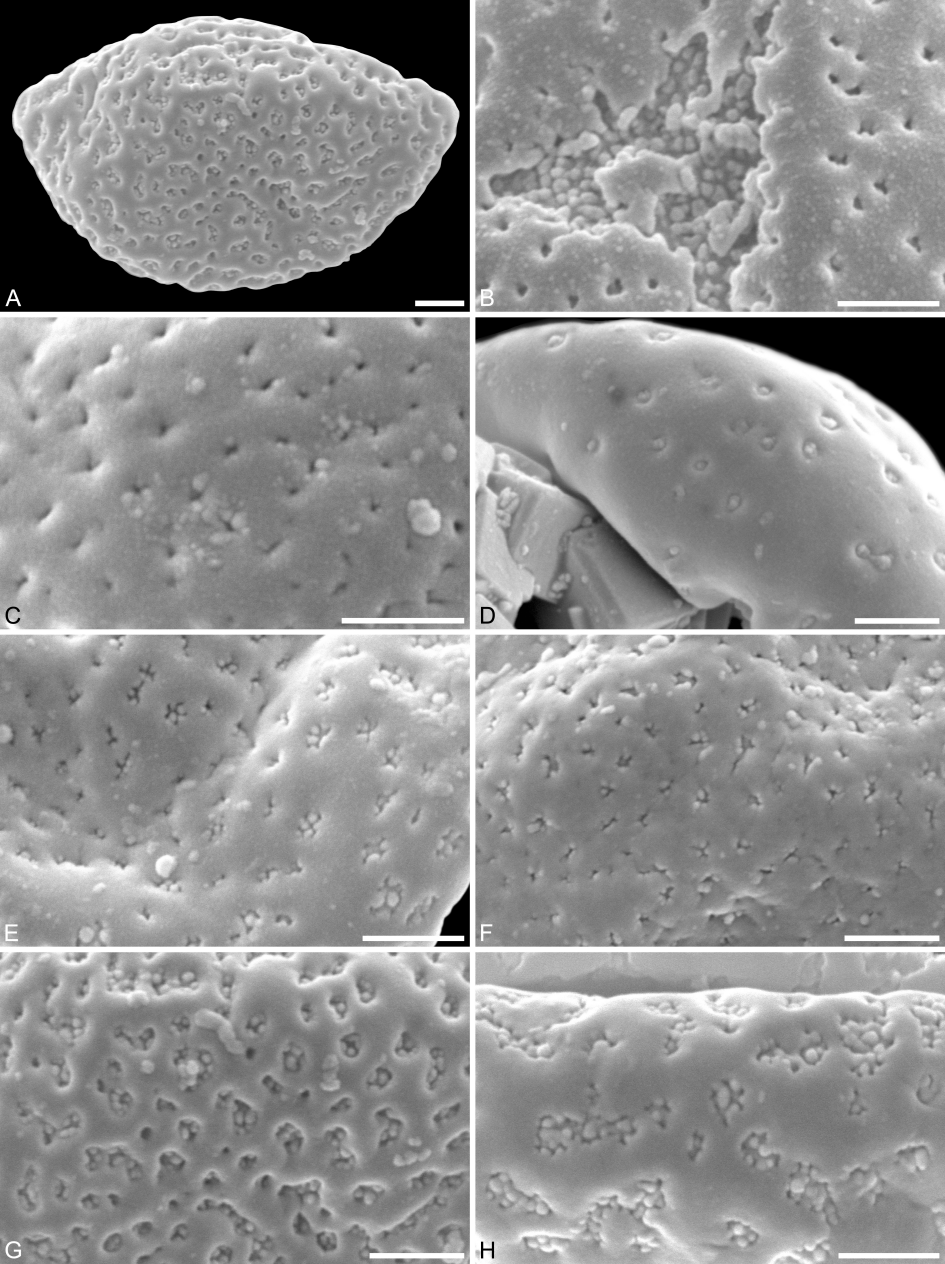
SEM micrographs of *Saururus aquilae* sp. nov. from the Upper Cretaceous (Campanian, 82–81 Ma) of Wyoming, western USA. (A) Paratype, IPUW 7513/109; pollen grain in proximal polar view, large perforations. (B) Close-up of Fig. 2H, showing densely packed columellae in an area of surface erosion. (C) Close-up of Fig. 2G, showing tiny perforations. (D) Paratype, IPUW 7513/110; close-up showing small circular perforations filled with columellae. (E) Close-up of Fig. 2D, showing irregular and lobate perforations and free-standing columellae. (F) Close-up of Fig. 2E, showing small irregular perforations and free-standing columellae. (G) Close-up of Fig. 3A, showing large circular to elliptic perforations and free-standing columellae. (H) Paratype, IPUW 7513/111; close-up showing large irregular perforations and free-standing columellae. Scale bars: 1 μm.

**Figure 4:**
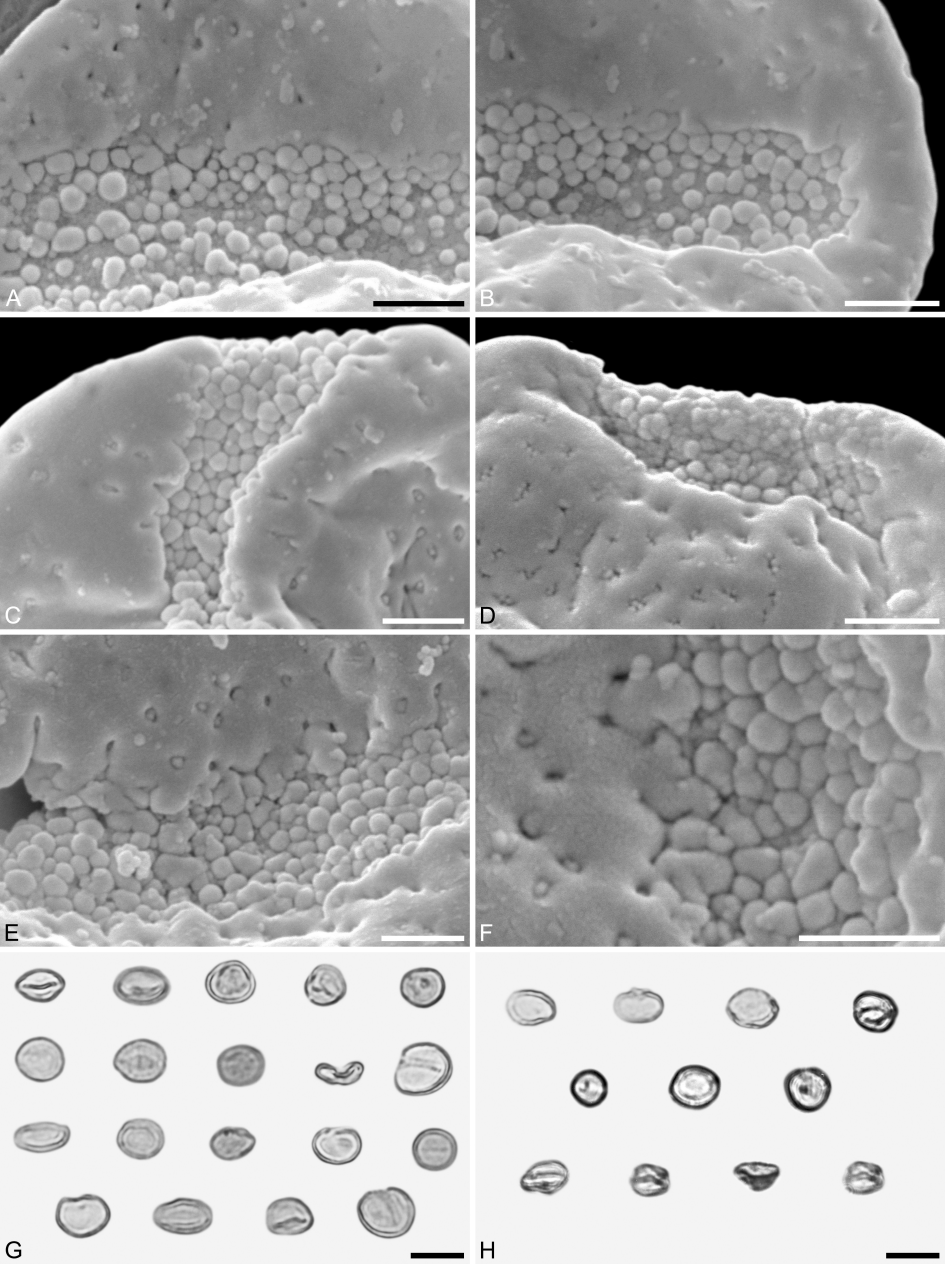
SEM and LM micrographs of *Saururus aquilae* sp. nov. (A–G; Campanian; Wyoming) and LM micrographs of *Saururus tuckerae* (H; middle Eocene; Princeton, B.C.) (A) Close-up of Fig. 2B, showing sulcus membrane, segregated microechini. (B) Close-up of Fig. 2B, showing sulcus membrane, segregated microechini. (C) Close-up of Fig. 2C, showing sulcus membrane, densely packed microechini. (D) Close-up of Fig. 2D, showing sulcus membrane, densely packed microechini. (E) Close-up of Fig. 2A (holotype), showing sulcus membrane. (F) Close-up of Fig. 4E (holotype), showing densely packed microechini. (G) *Saururus aquilae* sp. nov. pollen in LM. (H) *Saururus tuckerae* pollen in LM. Scale bars: Scale bars: 1 μm in (A–F), 10 μm in (G, H).

***Saururus aquilae* sp. nov. (Figs. 2–4)**

**Table:** 

**Holotype.** IPUW 7513/101 (Figs. 2A, 4E, 4F)
**Paratypes.** IPUW 7513/102–111 (Figs. 2B–2H, Fig. 3, 4A–4D, 4G, 4H)
**Type locality.** Elk Basin, Wyoming, United States.

**Stratigraphy and age.** Lettered Sands Member, Upper Eagle beds, Eagle Fm, Upper Cretaceous (Campanian); 82–81 Ma (Hicks, 1993; Van Boskirk, 1998).

**Species diagnosis.** Sculpture perforate, psilate to granulate; proximal face with about five perforations per μm^2^; perforations can have lobate outlines and up to six free-standing and/or protruding columellae; exine ≤400 nm and nexine <200 nm thick. All other pollen features that can be observed under LM and SEM as in the two modern species of the genus.

**Description.** Pollen, monad, shape oblate, form boat-like to globose, outline elliptic in equatorial and polar view; size very small, polar axis 3–5 μm long in SEM, equatorial diameter 6–11 μm in SEM; sulcate, sulcus with rounded ends (SEM); tectate; exine c. 400 nm thick, nexine c. 140 nm thick, nexine thinner than sexine (SEM); sculpture psilate in LM, perforate, psilate to granulate in SEM, 20–25 perforations per 4 μm^2^, perforations tiny to small, circular, elliptic, irregular, irregular elongated to lobate in outline, perforations fewer and smaller on distal polar face (SEM), perforations are characterized by 1–6 free-standing and/or protruding columellae, free-standing columellae at periphery of perforations or sometimes filling them completely (SEM); sulcus membrane microechinate, microechini mostly with blunt apex, microechini densely packed to segregated (SEM).

**Remarks.** The description is based on c. 50 individual dispersed pollen grains studied both in LM and SEM. The Cretaceous *S. aquilae* sp. nov. pollen grains are very similar to or indistinguishable from the Eocene pollen of *S. tuckerae* that has been found both *in situ* in inflorescences/flowers (Smith & Stockey, 2007a) and dispersed in the same sediments (Zetter, 2006; this study). The only differences are found in the sculpture of the sulcus membrane: in some grains of *S. aquilae* sp. nov., the microechini can be densely packed (Figs. 2A, 2C; Figs. 4C, 4E, 4F), whereas they are widely spaced in *S. tuckerae* and the two modern species of *Saururus* (Table 4; File S1). The pollen grains of both taxa are even smaller than pollen of Miocene (*S. stoobensis* sp. nov., below) and extant Saururaceae except for *Gymnotheca*. They show the same basic SEM sculpture ranging from perforate, psilate to granulate; a variation also seen in the Miocene pollen but not to the same degree in extant members of the Saururaceae. The main diagnostic feature distinguishing *S. aquilae* sp. nov. from the Cretaceous and *S. tuckerae* from the Miocene and modern species of the genus is their high density of perforations (≥20 per 4 μm^2^ on the proximal pollen face compared to ≤10 per 4 μm^2^ in *S. stoobensis* sp. nov., *S. cernuus*, and *S. chinensis*). Furthermore, they both show up to six free-standing/protruding columellae at the periphery of perforations compared to a maximum of four in extant species of the Saururaceae. Occasionally lobate perforations in addition to the more common circular, elliptical and irregular perforations represent a feature seen only in the fossil *Saururus* pollen and the extant *S. chinensis.* Exine and nexine in both taxa are slightly but consistently thinner than in extant Sauruaceae. The *S. aquilae* sp. nov. pollen grains differ from those of *Gymnotheca*, *Houttuynia*, and *Anemopsis*. Pollen grains of *Gymnotheca* differ from *S. aquilae* sp. nov. and fossil and extant *Saururus* by their prominently striate and nanoechinate SEM sculpture; their perforations are without free-standing/protruding columellae. *Houttuynia* pollen grains are considerably larger than pollen of *S. aquilae* sp. nov., and are unique within Saururaceae in having a microverrucate sulcus membrane; their exine is much thicker than in *S. aquilae* sp. nov. *Anemopsis* pollen grains have sulcus membranes that are echinate to rugulate, a feature not seen in any other fossil or extant Saururaceae.

**Derivation of name.** The species is named after the Eagle (lat. *aquila*) Fm.

**Figure 5:**
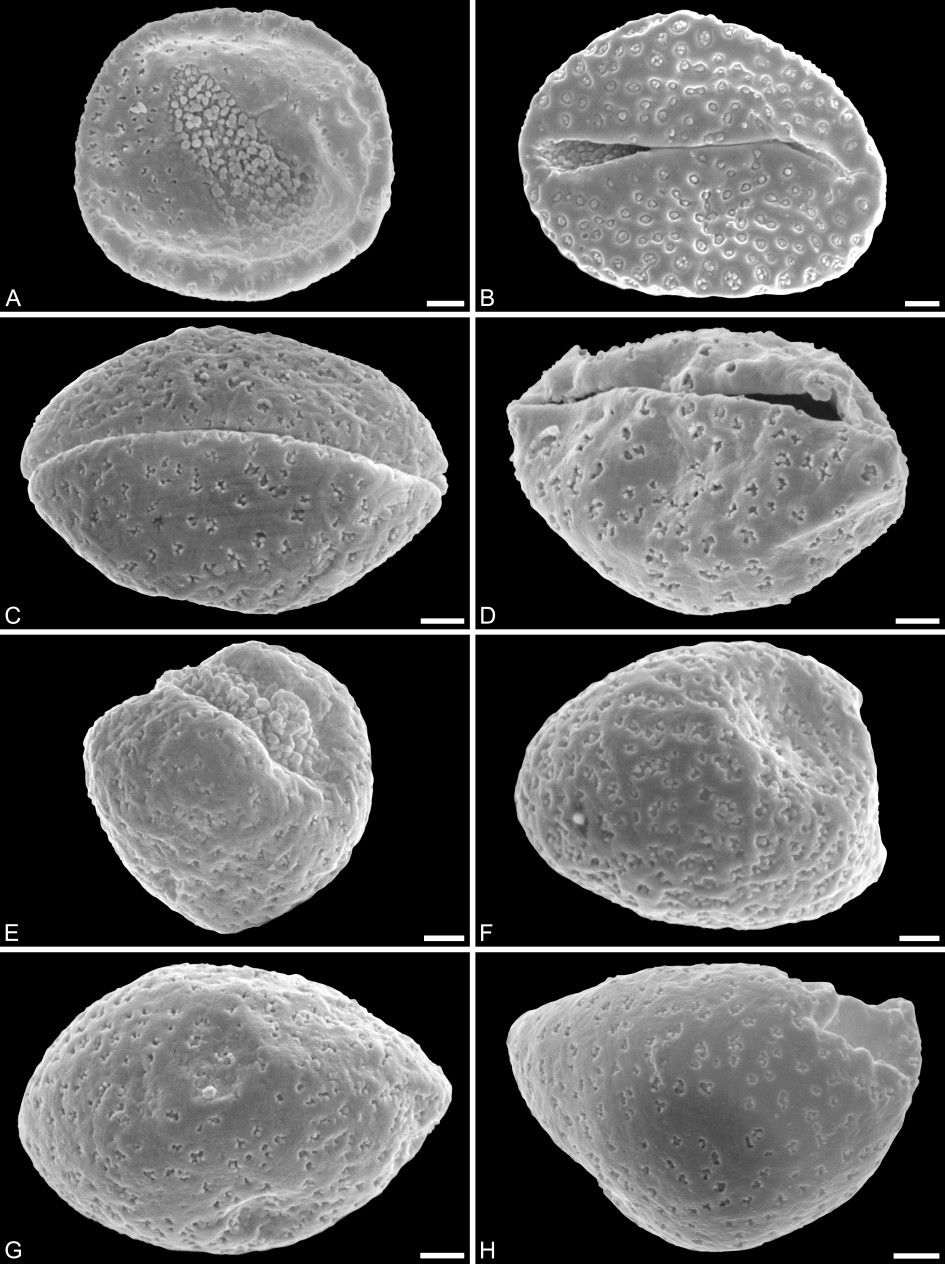
SEM micrographs of *Saururus tuckerae* pollen from the middle Eocene (c. 48 Ma) of Princeton, B.C., western Canada (A) Pollen grain (IPUW 7513/112) in distal polar view, showing sulcus, microechini segregated. (B) Pollen grain (IPUW 7513/113) in distal polar view, showing sulcus. (C) Pollen grain (IPUW 7513/114) in distal polar view, showing sulcus. (D) Pollen grain (IPUW 7513/115) in oblique equatorial view, showing sulcus. (E) Pollen grain (IPUW 7513/116) in equatorial view, showing sulcus and sulcus membrane. (F) Pollen grain (IPUW 7513/117) in proximal polar view. (G) Pollen grain (IPUW 7513/118) in proximal polar view. (H) Pollen grain (IPUW 7513/119) in proximal polar view. Scale bars: 1 μm.

**Figure 6:**
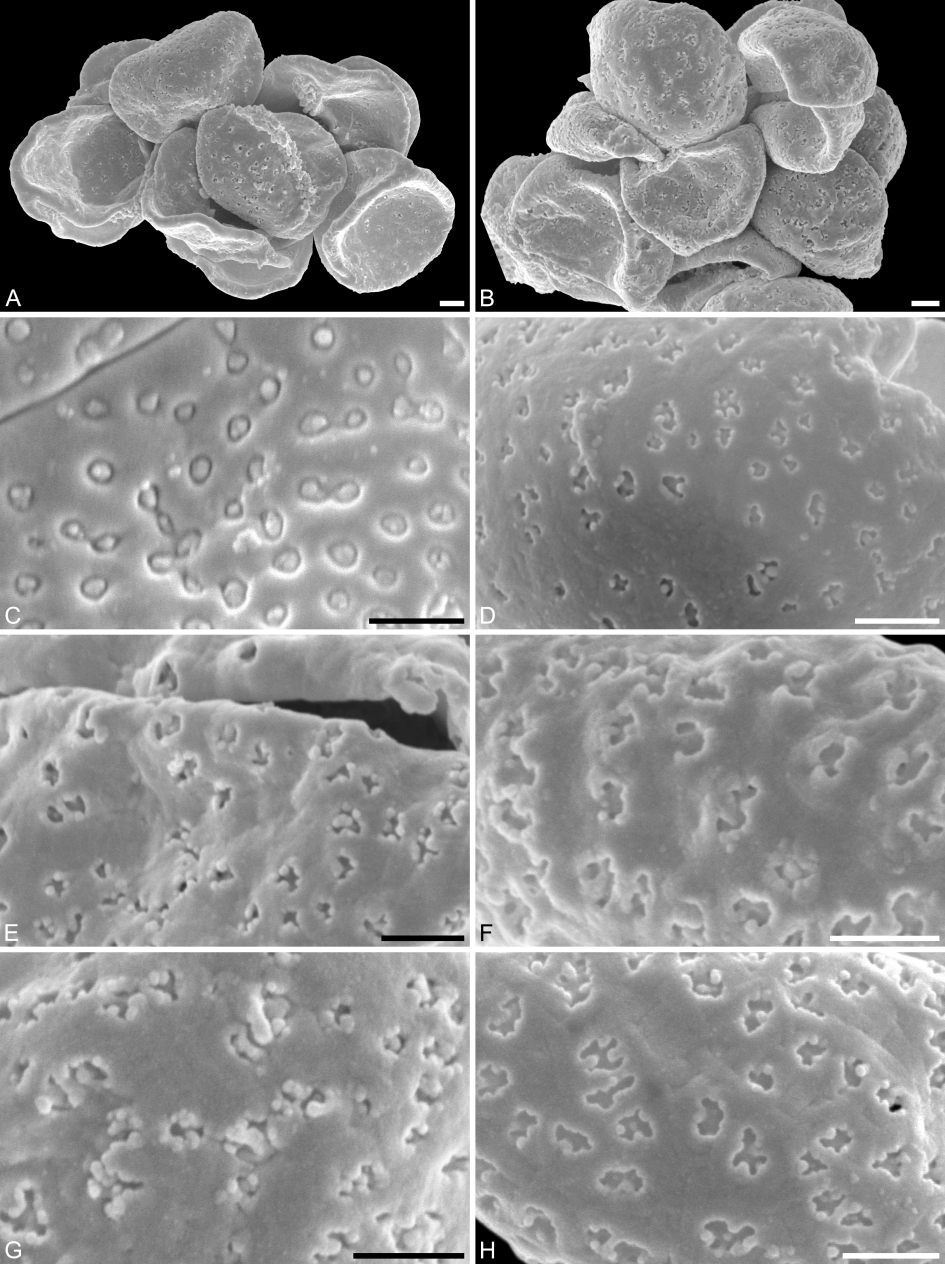
SEM micrographs of *Saururus tuckerae* pollen from the middle Eocene (c. 48 Ma) of Princeton, B.C., western Canada (A) Pollen grains preserved in a clump (IPUW 7513/120). (B) Pollen grains preserved in a clump (IPUW 7513/121). (C) Pollen grain, close-up of Fig. 5B, showing small circular to elliptic perforations filled with free-standing columellae. (D) Close-up of Fig. 5H, showing small irregular to lobate perforations. (E) Close-up of Fig. 5D, showing small irregular to lobate perforations, some with up to 6 free-standing columellae. (F) Pollen grain, IPUW 7513/122; close-up showing irregular to lobate perforations. (G) Close-up of Fig. 6B, showing irregular to lobate perforations with up free-standing columellae. (H) Close-up of pollen (IPUW 7513/123) grain showing irregular to lobate perforations. Scale bars: 1 μm.

**Figure 7:**
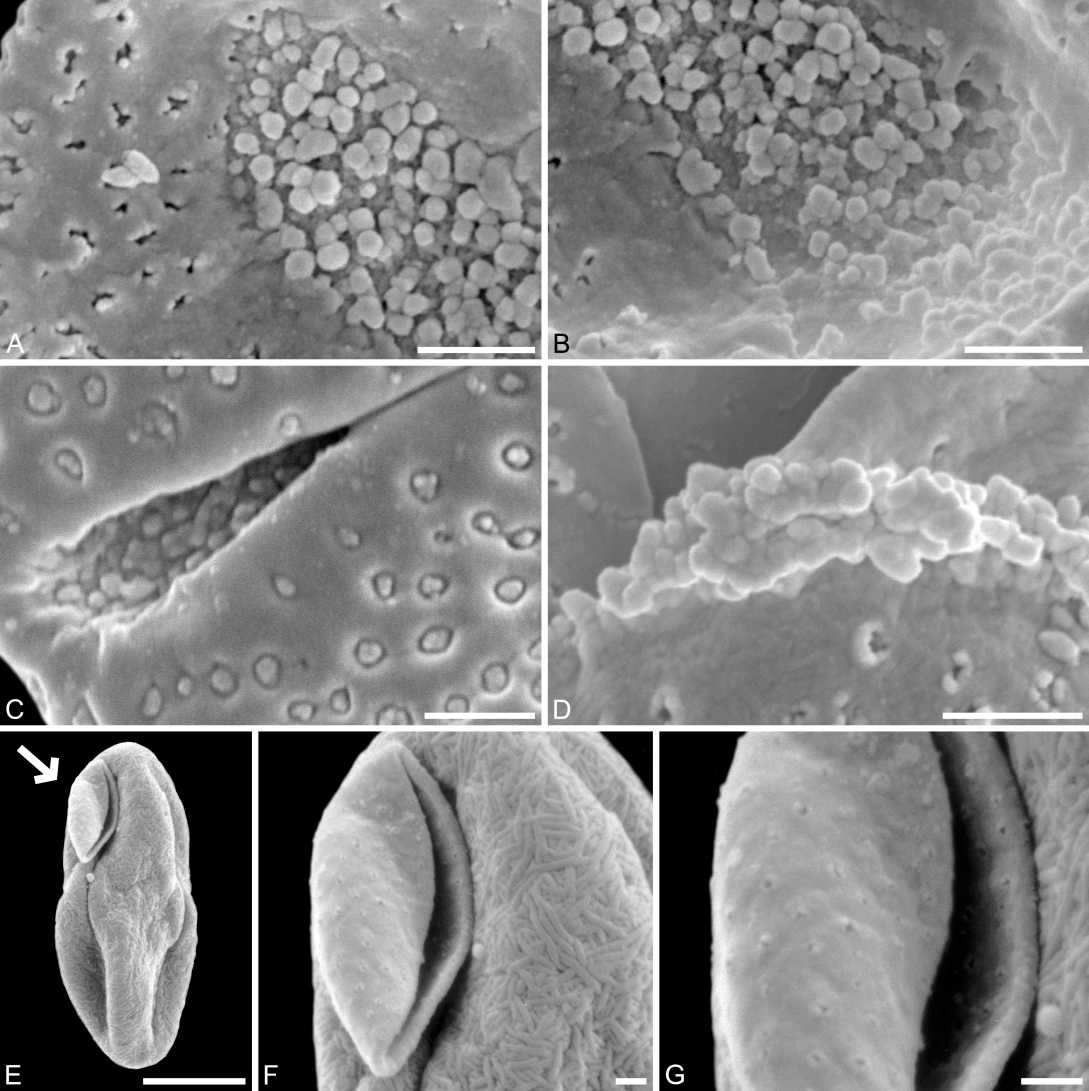
SEM micrographs of *Saururus tuckerae* (A–D; middle Eocene; Princeton, B.C.) and *Saururus stoobensis* sp. nov. from the Miocene Opencast clay pit, Stoob-Warasdorf-Forest, Burgenland, Austria (E–G). (A) Close-up of Fig. 5A, showing microechinate colpus membrane, microechini segregated. (B) Close-up of Fig. 5A, showing colpus membrane, microechini segregated. (C) Close-up of Fig. 5B, showing microechinate membrane. (D) Close-up of Fig. 6A, showing colpus membrane. (E) *Saururus stoobensis* sp. nov. holotype, IPUW 7513/124; grain (arrow) attached to a pollen grain of Apiaceae illustrating the size difference. (F) Close-up of Fig. 7E, overview of pollen. (G) Close-up of Fig. 7F, showing perforate sculpture with relatively few and tiny perforations. Scale bars: 1 μm in (A–D), (F), (G), 10 μm in (E).

**Saururus tuckerae *S.Y.Sm. & Stockey (*Figs. 4H*,*5*,*6*,*7A*–*7D*)***

**Table:** 

2007 “*Anemopsipollis* sp.” (nomen nudum)—Ferguson et al., pl. 2, figs. 5–8.
2007a *Saururus tuckerae*—Smith & Stockey, figs. 21, 22, 26, 29.
2007b *Saururus tuckerae*—Smith & Stockey, figs. 11A–11E, 12A–12C.

**Age.** Middle Eocene, c. 48 Ma (Moss, Greenwood & Archibald, 2005).

**Description.** Pollen, monad, shape oblate, form boat-like, outline elliptic in equatorial and polar view; size very small, polar axis 3–5 μm long in SEM, equatorial diameter 6–11 μm in SEM; sulcate, sulci with rounded ends (SEM); tectate; exine c. 370 nm thick, nexine c. 150 nm thick, nexine thinner than sexine (TEM); sculpture psilate in LM, perforate, psilate to granulate in SEM, 23–26 perforations per 4 μm^2^, perforations tiny to small, circular, elliptic, irregular, irregular elongated or lobate in outline, perforations fewer and smaller on distal polar face (SEM), perforations are characterized by 2–6 freestanding and/or protruding columellae, freestanding-columellae at periphery of perforations or sometimes filling it completely (SEM); sulcus membrane microechinate, microechini mostly with blunt apex, microechini segregated (SEM).

**Remarks.** The description is based on c. 200 individual dispersed pollen grains studied under LM and SEM, and compared with the *in-situ* grains figured in Smith & Stockey (2007a, 2007b). For additional remarks see remarks for *Saururus aquilae*.

***Saururus stoobensis* sp. nov. (Figs. 7E*–*7G*)***

**Table:** 

2007 “*Saururipollis* sp.” (nomen nudum)—Ferguson et al., Pl. 2, figs. 1–4 (same grain).

**Holotype.** IPUW 7513/124 (Figs. 7E–7G).

**Type locality.** Opencast clay pit, Stoob-Warasdorf-Forest, Burgenland, Austria.

**Age.** Miocene (Pannonian ?; = Tortonian to Messinian, c. 12–6 Ma; Klaus, 1982)

**Species diagnosis.** Sculpture perforate, psilate to granulate; perforations occasionally with lobate outlines. All other pollen features (size, form, sculpture of sulcus membrane, number of perforations per μm^2^) that can be observed under LM and SEM as in the two modern species of the genus.

**Table 4:** Pollen features of extinct (†) and extant Saururaceae. Unique (genus- or species-level) features in bold.

Species	Distibution/ provenance	E (μm; SEM)	P (μm; SEM)	Surface sculpture (SEM)	Sulcus membrane (SEM)	Perforations per 4 μm^2^ proximal face	Size and outline of perforations	Free-standing or protruding columellae	Exine thickness, mean (μm^2^)	Nexine thickness, mean (μm)
*Anemopsis californica*	SW US, NW Mexico	12–13	5–6	Perforate, granulate	**Echinate, rugulate**	7–9	Tiny; circular, elliptic, irregular	up to 6	0.47	0.20
*Gymnotheca chinenis*	SW and S China, Vietnam	9–10	4–5	Perforate, **striate, nanoechinate**	Microechinate	18–20	**Small**; circular, elliptic	**No**	0.55	0.18
*Gymnotheca involucrata*	S Sichuan (S China)	10–11	5–6	Perforate, **striate, nanoechinate**	Microechinate	17–19	**Small**; circular, elliptic	**No**	0.54	0.15
*Houttuynia cordata*	S and E Asia	13–14	8–9	Perforate, psilate	Microechinate	10–12	Tiny; circular, elliptic, irregular			
*Saururus cernuus*	E US	10–13	5–6	Perforate, granulate	Microechinate	7–9	Tiny; circular, elliptic, irregular	2–4	0.49	0.20
*Saururus chinensis*	S and E Asia	11–12	4–5	Perforate, psilate, **indistinctly rugulate**	Microechinate	6–8	Tiny; circular, elliptic, irregular, **lobate**	3–4	0.47	0.24
†*Saururus aquilae* sp. nov.	NW USA	6–11	3–5	Perforate, psilate to granulate	Microechinate, echini can be densely packed	20–25	Tiny to small; circular, elliptic, irregular, **irregular-elongated**, **lobate**	1–6	0.40	0.14
†*Saururus tuckerae*	SW Canada	6–11	3–5	Perforate, psilate to granulate	Microechinate, echini segregated	23–26	Tiny to small; circular, elliptic, irregular, **irregular-elongated**, **lobate**	2–6	0.37	0.15
†*Saururus stoobensis* sp. nov.	Austria	10–11	4–5	Perforate, psilate to granulate	Not observed	7–10	Tiny; circular, elliptic, irregular, **lobate**	2–4	Not observed	Not observed

**Notes.**

Abbreviations E equatorial diameter P polar axis SEM scanning-electron microscopy

Measurements and features from/based on Xi (1980), Takahashi (1986), Pontieri & Sage (1999), Sampson (2000), Furness, Rudall & Sampson (2002), Smith & Stockey (2007a, 2007b), Lu et al. (2015) and our own observations.

**Description.** Pollen, monad, shape oblate, form boat-like, outline elliptic in equatorial view; size very small, polar axis 4–5 μm long in SEM, equatorial diameter 10–11 μm in SEM; sulcate, sulci with rounded ends (SEM); tectate; sculpture psilate in LM, perforate, psilate to granulate in SEM, 7–10 perforations per 4 μm^2^, perforations tiny to small, circular, elliptic or irregular in outline, perforations fewer and smaller on distal polar face (SEM), perforations are characterized by 2–4 freestanding and/or protruding columellae, freestanding columellae at periphery of perforations (SEM).

**Remarks.** The Miocene *Saururus stoobensis* sp. nov. pollen is more similar to the pollen of extant *Saururus* (Table 4; File S1) than the Cretaceous and Eocene *Saururus* pollen. It is of similar size, has the same density of perforations and the same number of free-standing columellae. It slightly differs from both modern species in the variation of the sculpture seen in SEM, and the occasional occurrence of perforations with lobate outline, which can be found in *S. chinensis* and the older fossil taxa, but has so far not been observed in *S. cernuus* or other Saururaceae genera.

## Discussion

### Fossil records of Saururaceae on the backdrop of latest molecular age estimates (Massoni, Couvreur & Sauquet, 2015b)

**Figure 8:**
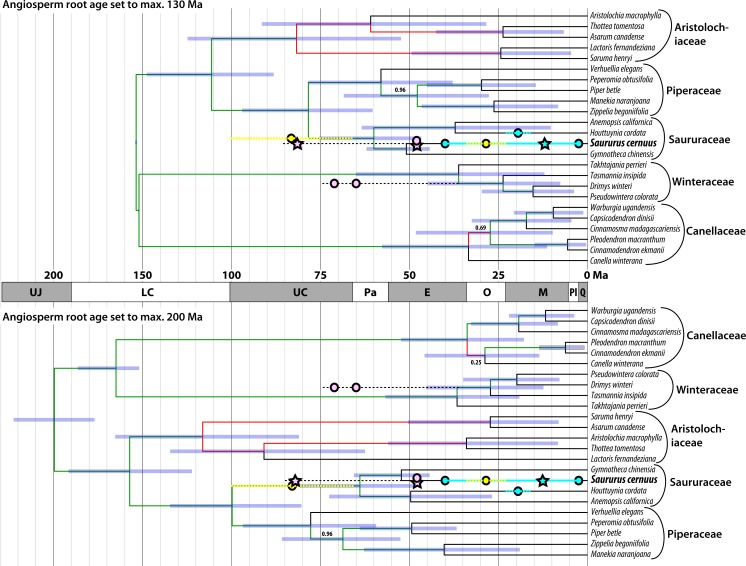
Mapping of the fossil record (circles, stars) of Saururaceae on dated phylogenies (Bayesian uncorrelated clock; included in Massoni, Couvreur & Sauquet, 2015a). Oldest fossils of the Winteraceae are shown for comparison. Pink: North American fossils; cyan: western Eurasian (Central Europe to western Siberia) fossils; yellow: East Asian fossils; stars: fossil pollen described here. Blue bars represent the 95% highest posterior density (HPD) intervals of the minimum age and maximum age scenarios; node heights are averages (medians are indicated by deep blue bars in the HPD intervals). Branch labels show posterior probabilities (PP) < 1.0 (all other branches have PP = 1.00), red branches highlight topological conflict between the chronograms (probably due to incomprehensive Bayesian runs getting stuck in local suboptima, since all analyses were based on the same data set).

Until now the fossil record of Saururaceae has been confined to the Cenozoic except for wood remains from the Late Cretaceous of Japan described a century ago (Stopes & Fujii, 1910). Most specimens have been linked to the extant genus *Saururus* (Table 2). Figure 8 shows the fossil record in comparison to the magnoliid subtree that includes the Saururaceae, extracted from the dated trees provided by Massoni, Couvreur & Sauquet (2015a). The fossil pollen *S. aquilae* sp. nov. described here from the middle Late Cretaceous (Campanian) of Wyoming, conflicts with the youngest dating estimates, which infer a Late Cretaceous to Paleocene root age for the Saururaceae. Under the oldest age scenario (Massoni, Couvreur & Sauquet, 2015a, 2015b), the Wyoming pollen falls (time-wise) in the (arithmetic) middle between the Saururaceae root and crown divergence ages. On the backdrop of the dating estimates, *S. aquilae* could be the pollen produced by a potential precursor of all extant Saururaceae genera (hypothesis 1). Hypothesis 1 would fit also with the interpretation of the Cretaceous fossil wood described as *Saururopsis nipponensis* from Japan (Stopes & Fujii, 1910). Although being more similar to wood of *Saururus*, Stopes and Fujii state that some features are reminiscent of *Houttuynia*, which belongs to the second lineage of extant Saururaceae (e.g., Massoni, Forest & Sauquet, 2014), and discussed the possibility that the wood comes from an ancestral member of the family. On the other hand, the tip ages are poorly constrained (likely too young) using Massoni, Couvreur & Sauquet’s (2015b) dataset, who focussed on (much) deeper nodes. Hence, *S. aquilae* sp. nov. could represent an early member of the *Saururus*-lineage (hypothesis 2). Notably, the age of *S. aquilae* sp. nov. is close to the lower boundary of the highest posterior density (HPD) intervals for the older age scenarios (Fig. 8; Massoni, Couvreur & Sauquet, 2015a). Being interested in large-scale magnoliid processes, Massoni, Couvreur & Sauquet (2015b) did not use any age priors from within the Piperales subtree (Massoni, Doyle & Sauquet, 2015) and relied on relatively slow-evolving gene regions. It is a common observation that divergence ages towards the leaves of a tree tend to be (severely) underestimated in studies using large datasets when compared to focused studies that rely on ingroup constraints. Typically, the latter are in better agreement with the fossil record, as e.g., in the case of the Fagaceae (Hubert et al., 2014; Grímsson et al., 2015; Grímsson et al., 2016a; Renner et al., 2016). A similar observation can be made in the sister group of the Piperales, the Canellales. Figure 8 also shows the oldest records of the Winteraceae, which are much older than the age estimates by Massoni, Couvreur & Sauquet (2015a, 2015b). Studies focusing on either Canellaceae (Müller et al., 2015) or Winteraceae (Marquínez et al., 2009; Thomas et al., 2014), using different sets of age priors including Canellaceae and Winteraceae crown group fossils, obtained (much) older ages than the oldest age scenario (angiosperm root fixed to max. 200 Ma) in the set of analyses performed by Massoni, Couvreur & Sauquet (2015b). Differences range from at least 13 Ma for the Canellaceae and Winteraceae roots to more than 40 Ma for close-to-tips nodes.

**Figure 9:**
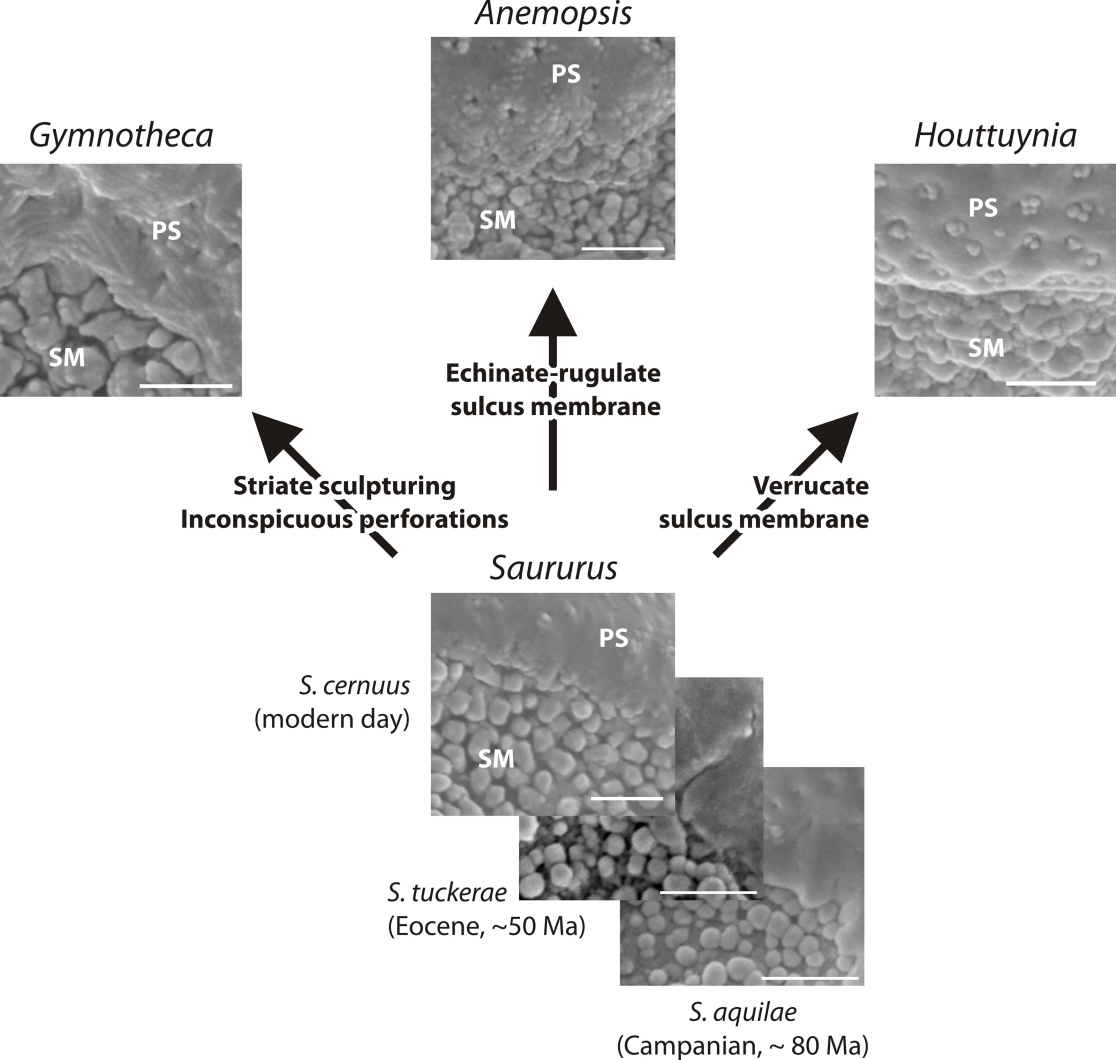
Hypothetical evolution of Saururaceae pollen. The fossil and extant *Saururus* show a morphology that may be primitive within the family: all other genera differ by one or two unique, putatively derived traits. Abbreviations: PS, (normal) pollen surface; SM, sulcus membrane. Scale bars = 1 μm.

Based on pollen morphology, neither hypotheses can be rejected. *Saururus aquilae* sp. nov. is essentially indistinguishable from pollen linked to the c. 30 Ma younger *S. tuckerae*, and all modern Saururaceae pollen types (≥35 Ma younger) differ only in a few characters (Table 4; File S1). The differences are often expressed as a range of variability (such as outlines of perforations or sculpture of the pollen surface). This demonstrates that pollen morphology is a very conservative trait in the lineage, and provides an argument for hypothesis 2 that *S. aquilae* sp. nov. was produced by an early member of the genus *Saururus.* On the other hand, the extant six species are clearly only the last survivors of a once more widespread group (Table 1), and may be unrepresentative regarding the actual variation in each generic lineage and the family over time. The pollen of *Saururus* may simply be primitive within the family. Ancestral members of the Saururaceae including precursors of all modern genera may have produced essentially the same pollen (hypothesis 1), whereas pollen morphologies of the extant species of the other genera are more derived (Fig. 9).

### The importance of an in-depth analysis of the dispersed pollen record

Pollen of Saururaceae is very small (literally, using the size categories of Hesse et al., 2009), and has probably been overlooked or ignored in many palynological studies (see also Smith & Stockey, 2007b). A major problem that directly affects the recovery of Saururaceae pollen is that the standard (LM) paleopalynological approach is to sieve the sediment with 10 μm sieves, which means that most if not all Saururaceae pollen will be lost. Hence, the lack of Saururaceae pollen all over the Northern Hemisphere may be in part a sieving artefact. The single grain method using a combination of LM and SEM imaging (e.g., Zetter, 1989) is a time-consuming approach, but as demonstrated here the information obtained can be highly beneficial to other botanical disciplines such as *(i)* molecular dating by providing new/alternative age priors (e.g., Hubert et al., 2014) and *(ii)* the study of historical biogeography by providing actual evidence for the occurrence of a certain lineage at a certain time in a certain place (e.g., Denk, Grímsson & Zetter, 2010; Grímsson, Zetter & Hofmann, 2011; Grímsson et al., 2015; Grímsson et al., 2016a). A main advantage of pollen for assessing past distribution is its high evolutionary conservatism across long periods of time. For the Saururaceae the data presented here and the *in-situ* grains showed by Smith & Stockey (2007a) prove that the main characteristics of Saururaceae pollen (Smith & Stockey, 2007b; this study) have remained essentially unchanged for over 80 Ma. This is not an exception; *Fagus,* castaneoid and cornalean pollen can be traced back at least to the Danian of western Greenland (Manchester, Grímsson & Zetter, 2015; Grímsson et al., 2016a), and is part of a very rich pollen flora covering at least 32 families of angiosperms (Grímsson et al., 2016b); castanoid pollen of the sister clade of *Fagus* (all other Fagaceae) has been found in the same sample as the Saururaceae pollen described here (Grímsson et al., 2016a) in addition to asteroid families such as the Araliaceae and Oleaceae (Manchester, Grímsson & Zetter, 2015). Friis, Crane & Pedersen (2011) consistently and repeatedly express their concern regarding the affiliation of many (macro) fossils of the Cretaceous fossil record with angiosperm taxa. For instance, regarding *Saururopsis niponensis*, the Late Cretaceous wood from Hokkaido (Stopes & Fujii, 1910), they state that “the relationships of this material require further study” (Friis, Crane & Pedersen, 2011, p. 248). We agree, and advocate the use of comprehensive, in-depth studies of the dispersed and *in-situ* pollen record using the combination of LM and SEM imaging on the same, single grain to fill the many gaps obscuring the origin of the angiosperms, their Cretaceous diversity and spatial distribution, and the roots of the modern lineages and their precursors (e.g., Doyle, Hotton & Ward, 1990; Takahashi, 1997; Zetter, Hesse & Huber, 2002; Hofmann & Zetter, 2007; Hofmann & Zetter, 2010; Grímsson, Zetter & Hofmann, 2011; Hofmann et al., 2011; Grímsson et al., 2014; Grímsson et al., 2016a; Mendes et al., 2014; Manchester, Grímsson & Zetter, 2015).

##  Supplemental Information

10.7717/peerj.3434/supp-1 File S1 Pollen morphology of extant Saururaceae Micrographs of extant Saururaceae pollen under light (LM)- and scanning electron microscopy (SEM).

10.7717/peerj.3434/supp-2 Table S1 Herbarium specimens used for this study

## Additional Information and Declarations

### New Species Registration

The following information was supplied regarding the registration of a newly described species: Publication LSID:65F8D73C-E7B9-4AF9-868B-567EB53276C7
http://fossilplants.info/publications/65F8D73C-E7B9-4AF9-868B-567EB53276C7. *Saururus aquilae*, fossil pollen species LSID, IFPNI: 9999EA0B-85E2-4BD7-9F57-2AB412345314
http://fossilplants.info/names/9999EA0B-85E2-4BD7-9F57-2AB412345314. *Saururus stoobensis*, fossil pollen species LSID, IFPNI: 854E525C-CE8E-438E-AE86-B3536ADCE3DB
http://fossilplants.info/names/854E525C-CE8E-438E-AE86-B3536ADCE3DB.
