# Supplementary material for: Tiny pollen grains: first evidence of Saururaceae from the Late Cretaceous of western North America
Source: PeerJ. 2017 Jun 13;5:e3434. doi: 10.7717/peerj.3434 (PMC5472062; doi:10.7717/peerj.3434)
Supplement: File S1 — Micrographs of extant Saururaceae pollen under light (LM)- and scanning electron microscopy (SEM). [file peerj-05-3434-s001.pdf]

Supplementary File S1

Pollen morphology of extant Saururaceae

CONTENT

This supplement file provides micrographs of extant Saururaceae pollen under light (LM)- and scanning electron microscopy (SEM).

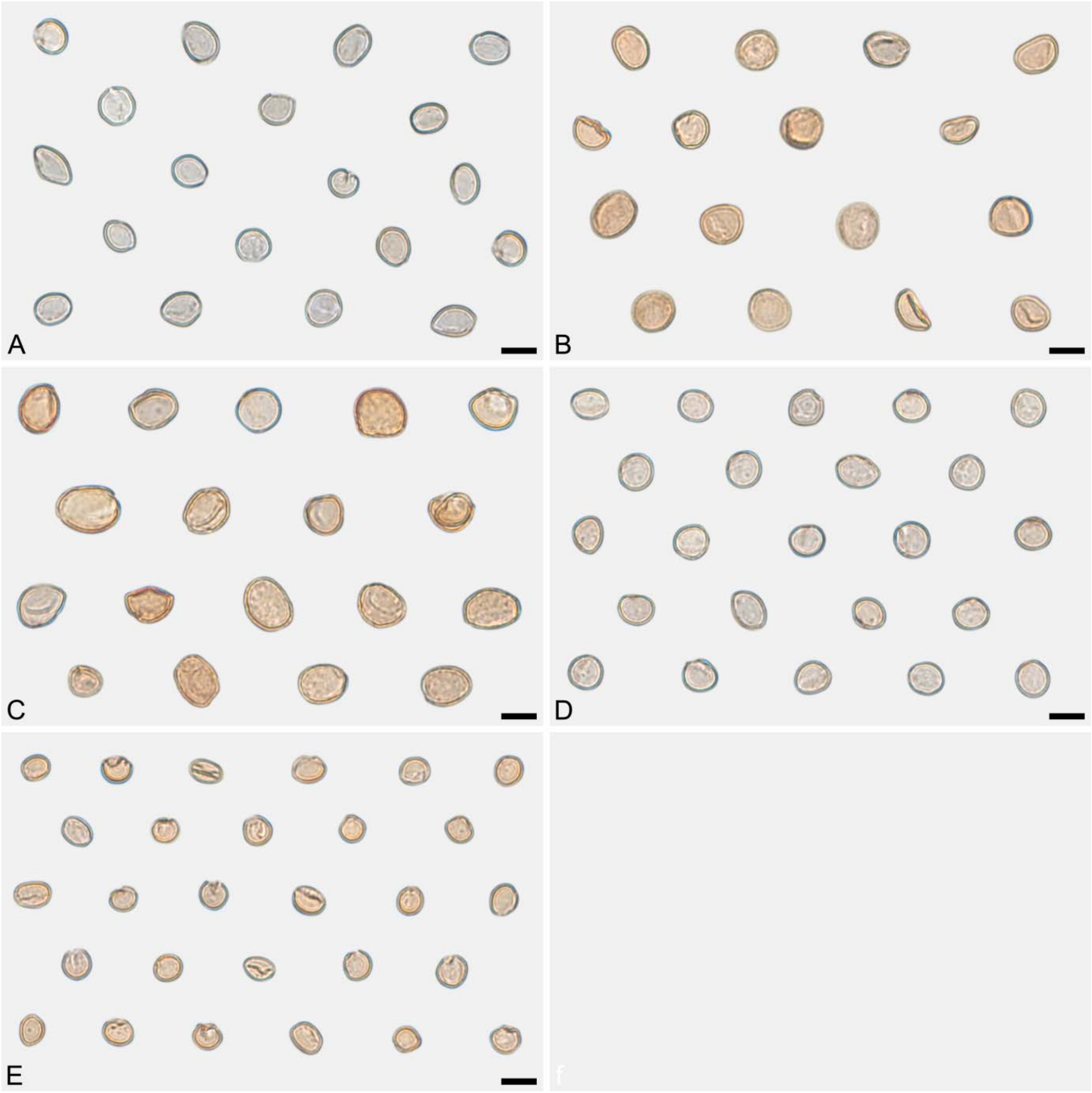

Figure S1. LM micrographs of extant Saururaceae.

A) *Anemopsis californica* Hook & Arn. [WU: Mexico, Baja California, Rancho La Suerte; coll. R.F. Thorne, s.n.]. **B)** *Gymnotheca chinensis* Decne. [WU 0039758]. **C)** *Houttuynia cordata* Thunb. [WU 0039759; WU 0062658]. **D)** *Saururus cernuus* L. [WU 0039743]. **E)** *Saururus chinensis* (Lour.) Baill. [WU 0039752]. Scale bars: 10 µm.

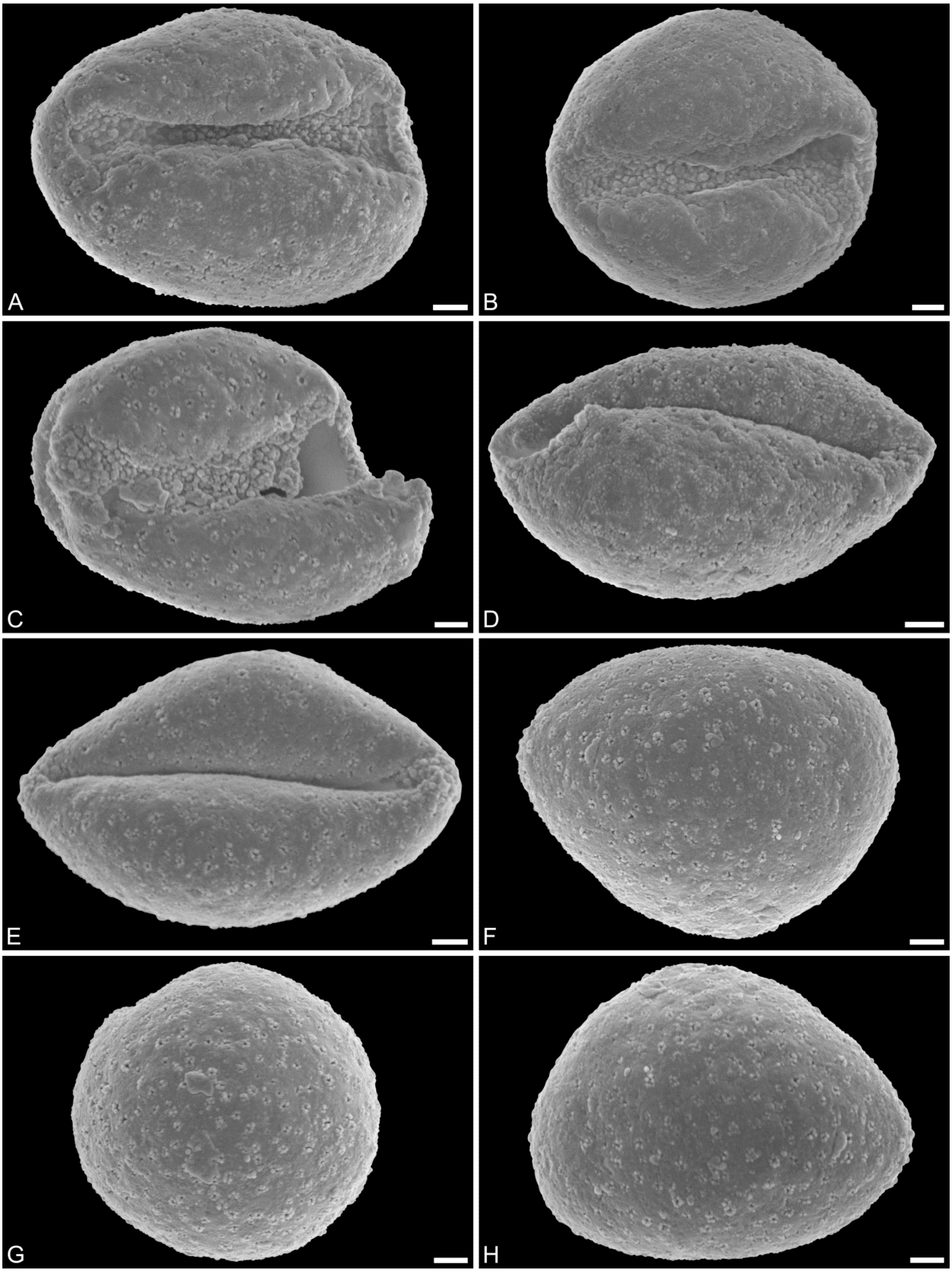

Figure S2. SEM micrographs of *Anemopsis californica* Hook & Arn. [WU: Mexico, Baja California, Rancho La Suerte; coll. R.F. Thorne, s.n.]. IPUW 7513/126.

A–C) Pollen in distal polar view, showing sulcus and sulcus membrane.

D–E) Pollen in distal polar view, showing sulcus area.

F–H) Pollen in proximal polar view.

Scale bars: 1 µm.

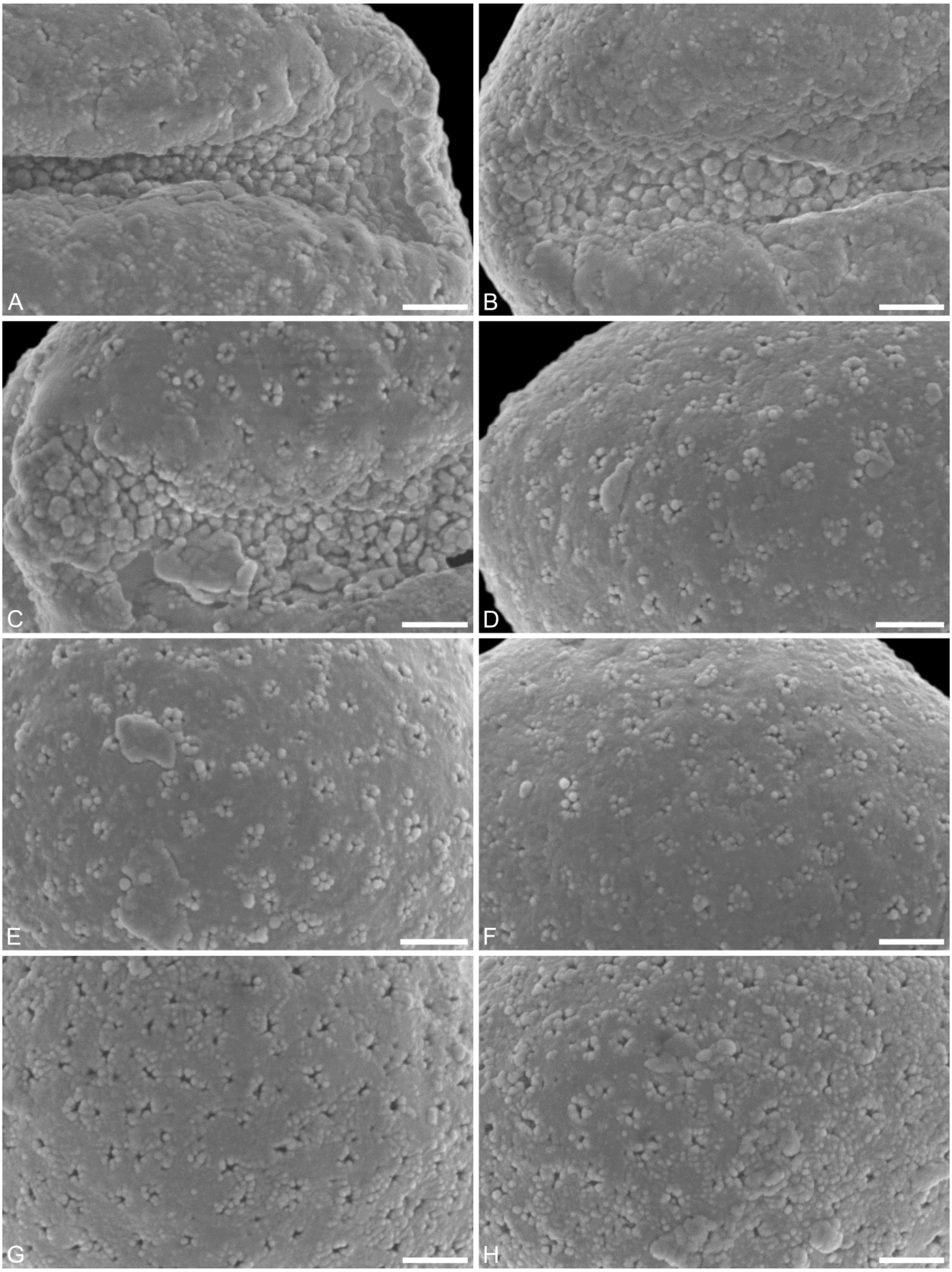

Figure S3. SEM micrographs of *Anemopsis californica* Hook & Arn. [WU: Mexico, Baja California, Rancho La Suerte; coll. R.F. Thorne, s.n.]. IPUW 7513/126.

A–C) Close-ups of sulcus membrane.

D–H) Close-ups of sculpture on proximal side of pollen.

Scale bars: 1 µm.

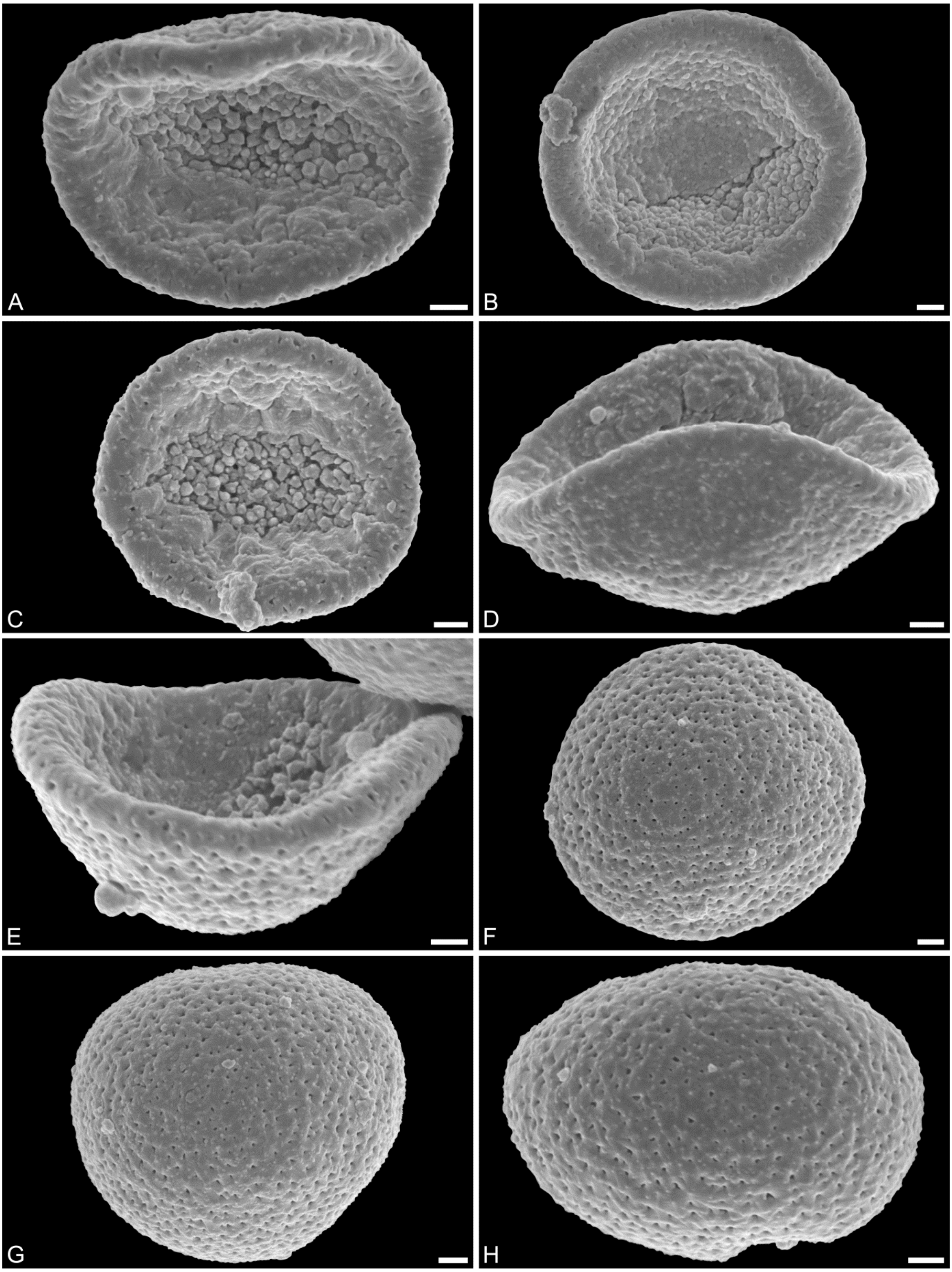

Figure S4. SEM micrographs of *Gymnotheca chinensis* Decne. [WU 0039758]. IPUW 7513/127.

A–C) Pollen in distal polar view, showing sulcus and sulcus membrane.

D–E) Pollen in equatorial view.

F–H) Pollen in proximal polar view.

Scale bars: 1 µm.

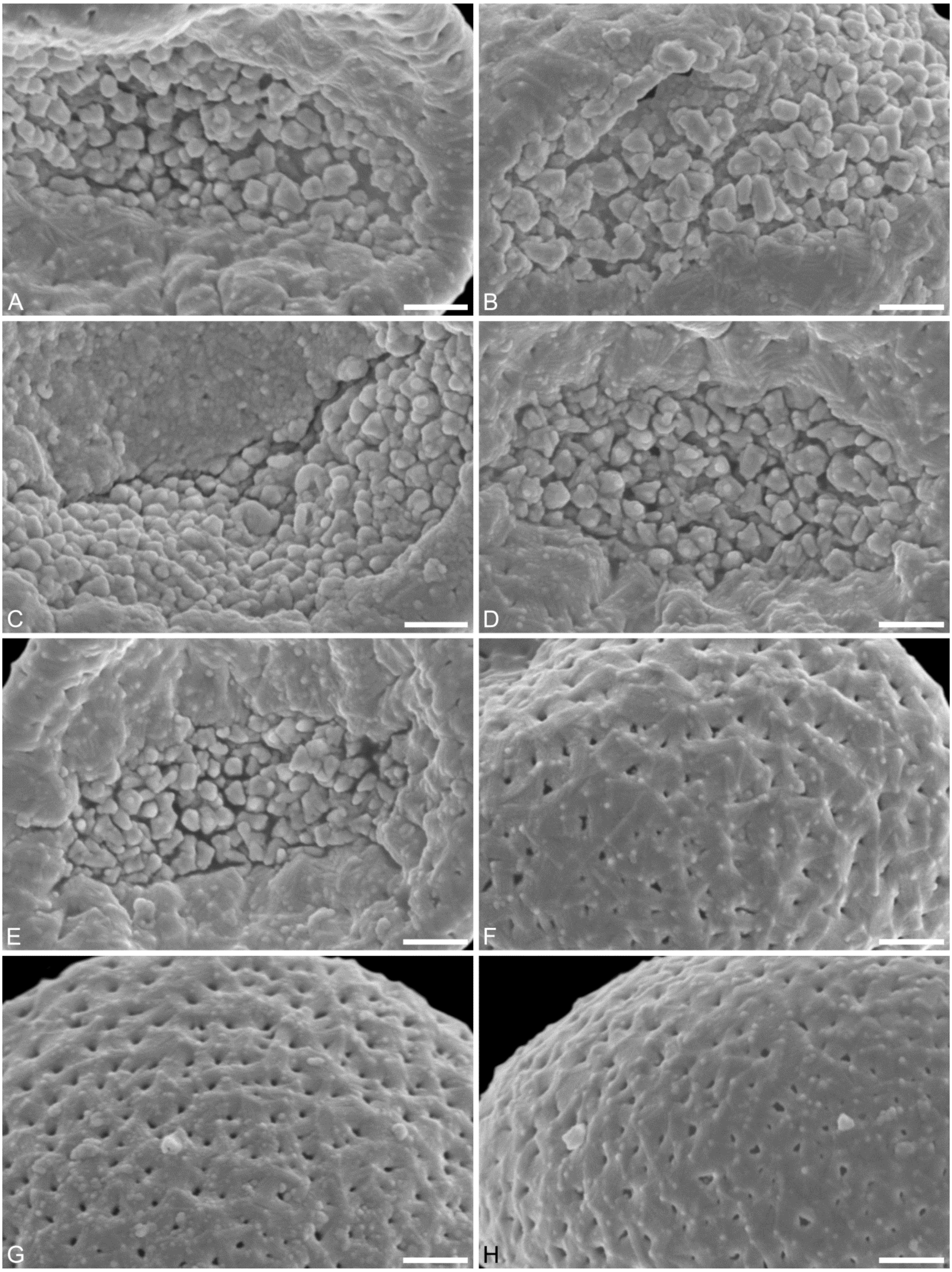

Figure S5. SEM micrographs of *Gymnotheca chinensis* Decne. [WU 0039758]. IPUW 7513/127.

A–E) Close-ups of sulcus membrane.

F–H) Close-ups of sculpture on proximal side of pollen.

Scale bars: 1 µm.

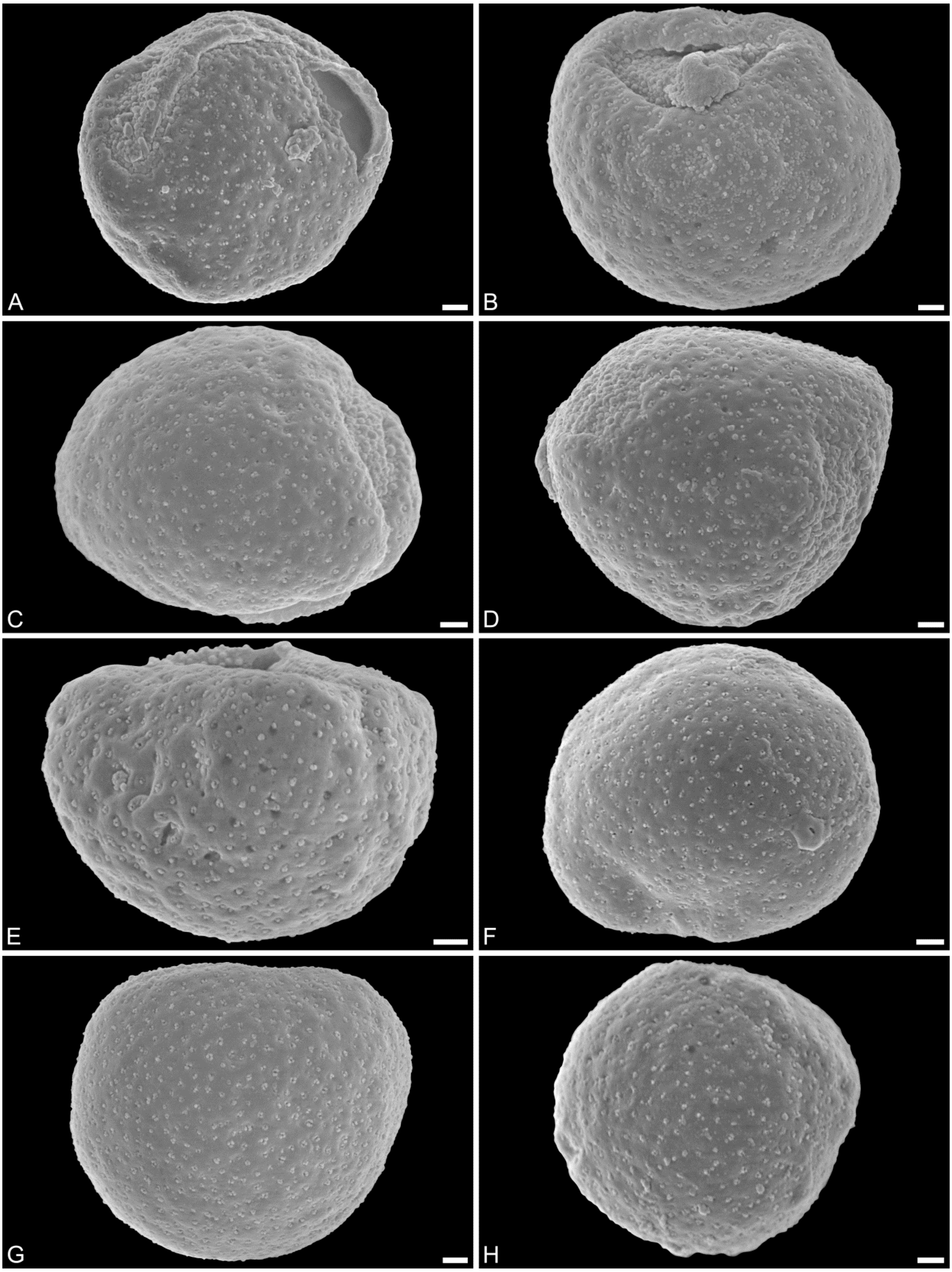

Figure S6. SEM micrographs of *Houttuynia cordata* Thunb. [WU 0039759]. IPUW 7513/128.

A–D) Pollen in distal polar or oblique view, showing sulcus and sulcus membrane.

E) Pollen in equatorial view.

F–H) Pollen in proximal polar view.

Scale bars: 1 µm.

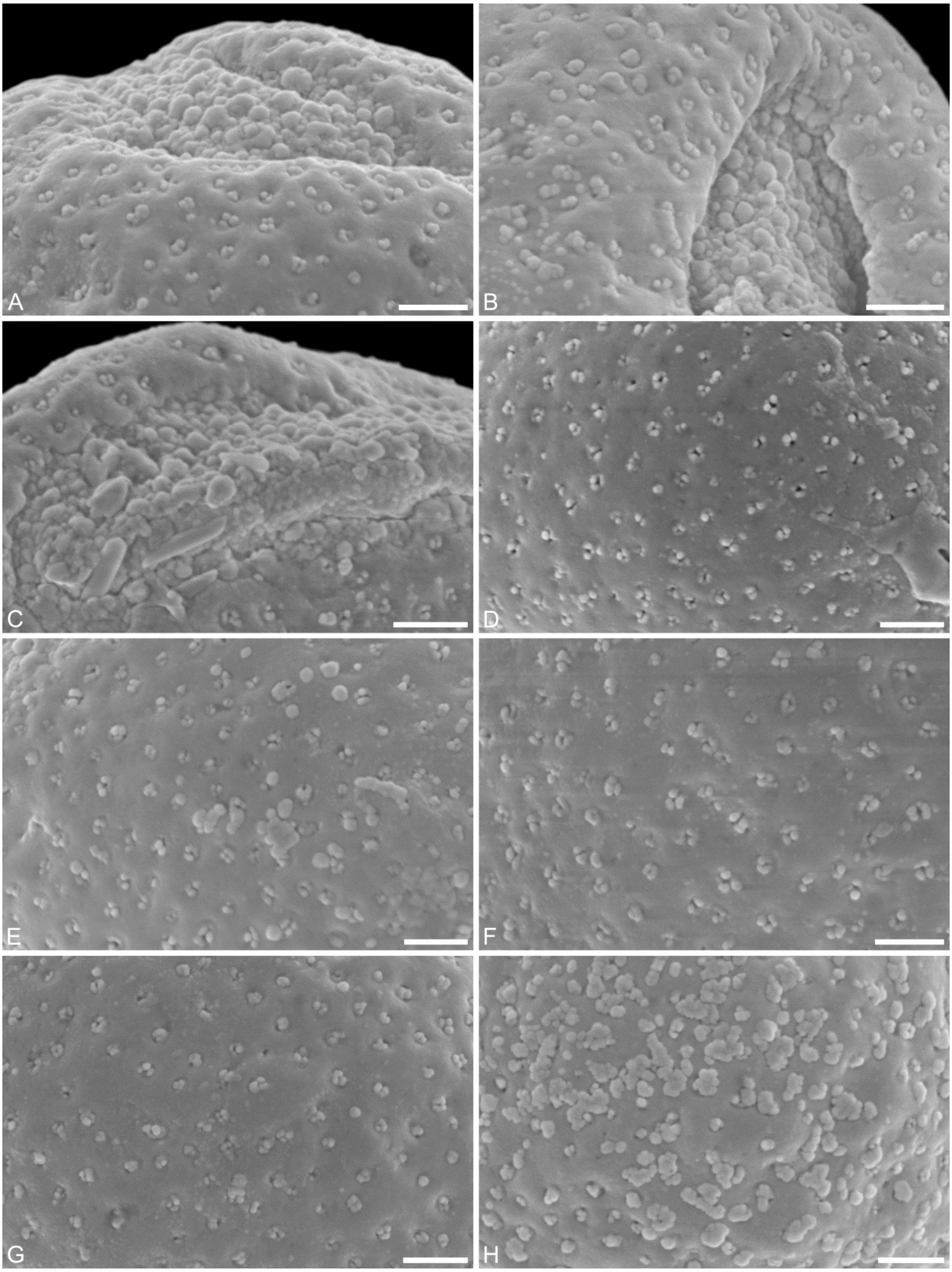

Figure S7. SEM micrographs of *Houttuynia cordata* Thunb. [WU 0039759]. IPUW 7513/128.

A–C) Close-ups of sulcus membrane.

D–H) Close-ups of sculpture on proximal side of pollen.

Scale bars: 1 µm.

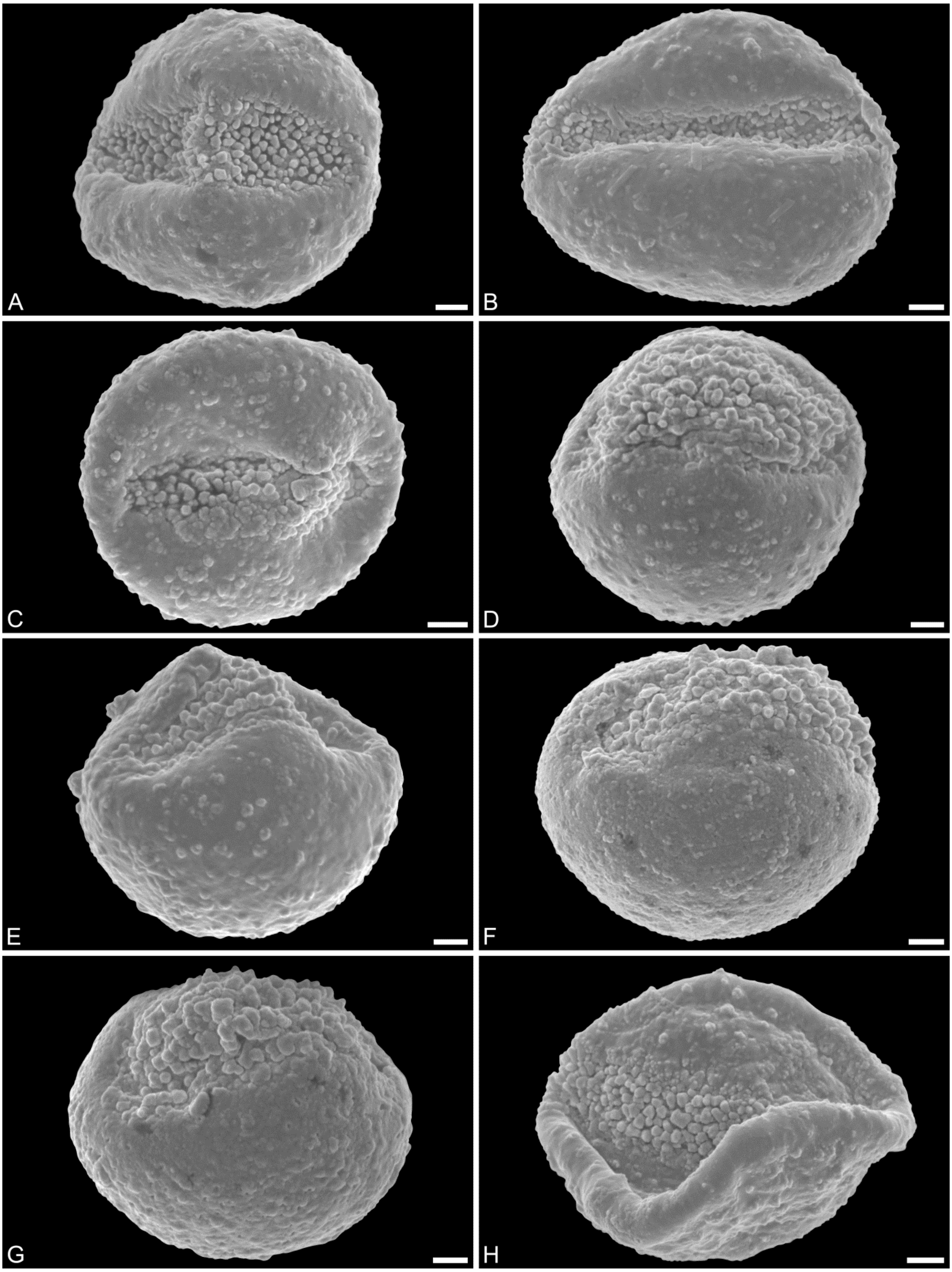

Figure S8. SEM micrographs of *Saururus cernuus* L. [WU 0039743]. IPUW 7513/129.

A–C) Pollen in distal polar view, showing sulcus and sulcus membrane.

D–H) Pollen in equatorial view.

Scale bars: 1 µm.

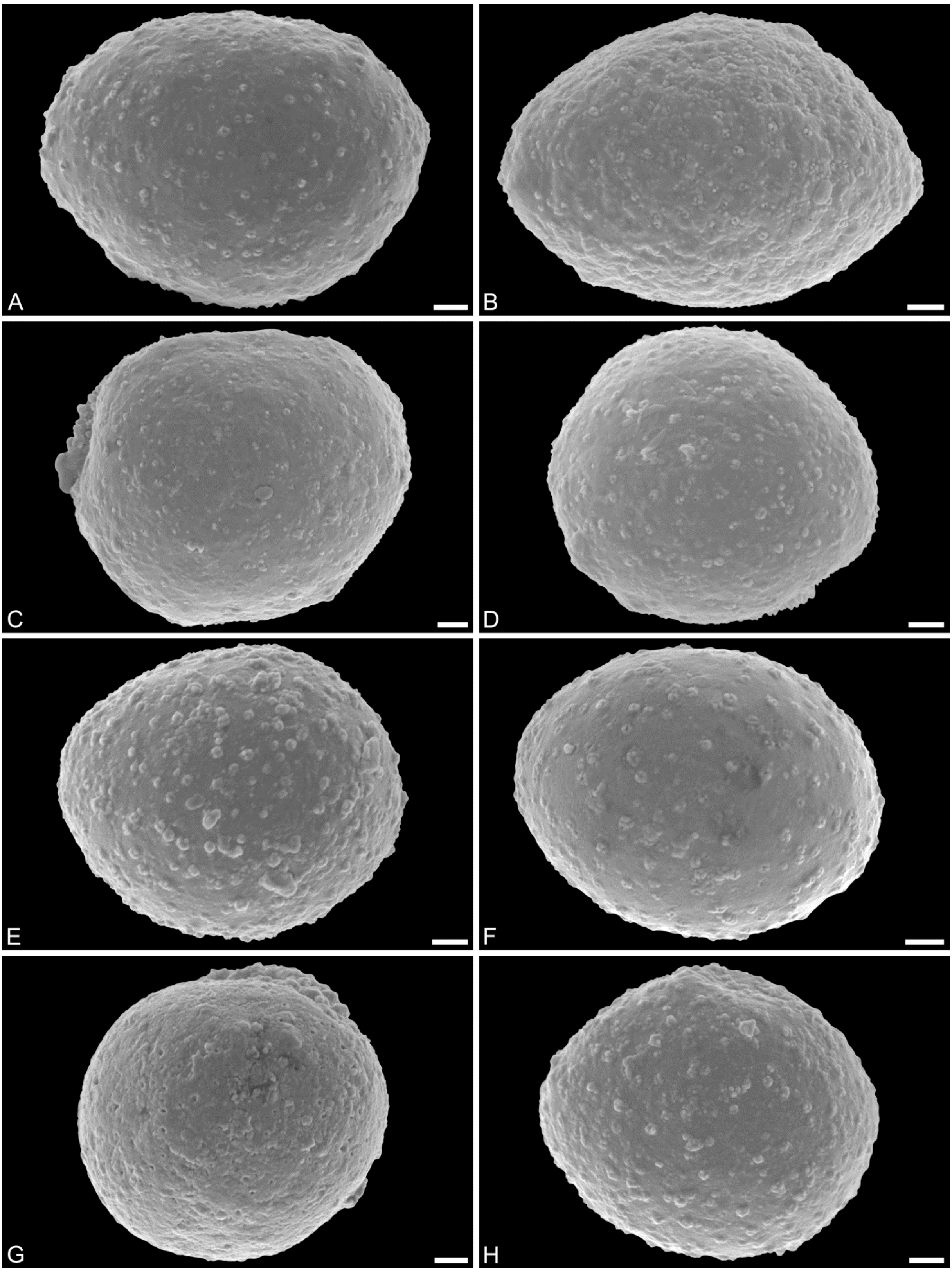

Figure S9. SEM micrographs of *Saururus cernuus* L. [WU 0039743]. IPUW 7513/129.

A–H) Pollen in proximal polar view.

Scale bars: 1 µm.

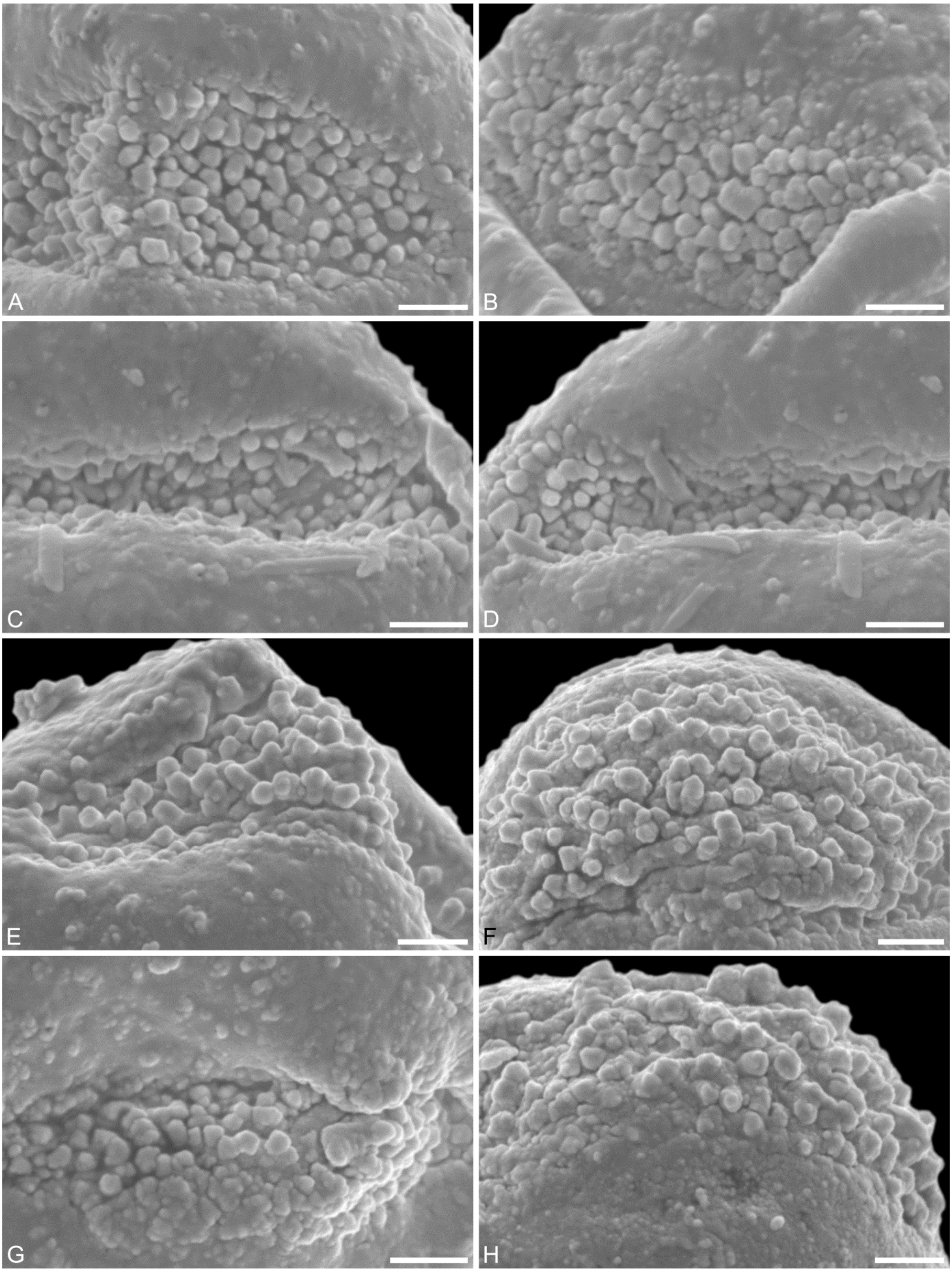

Figure S10. SEM micrographs of *Saururus cernuus* L. [WU 0039743]. IPUW 7513/129.

A–H) Close-ups of sulcus membrane.

Scale bars: 1 µm.

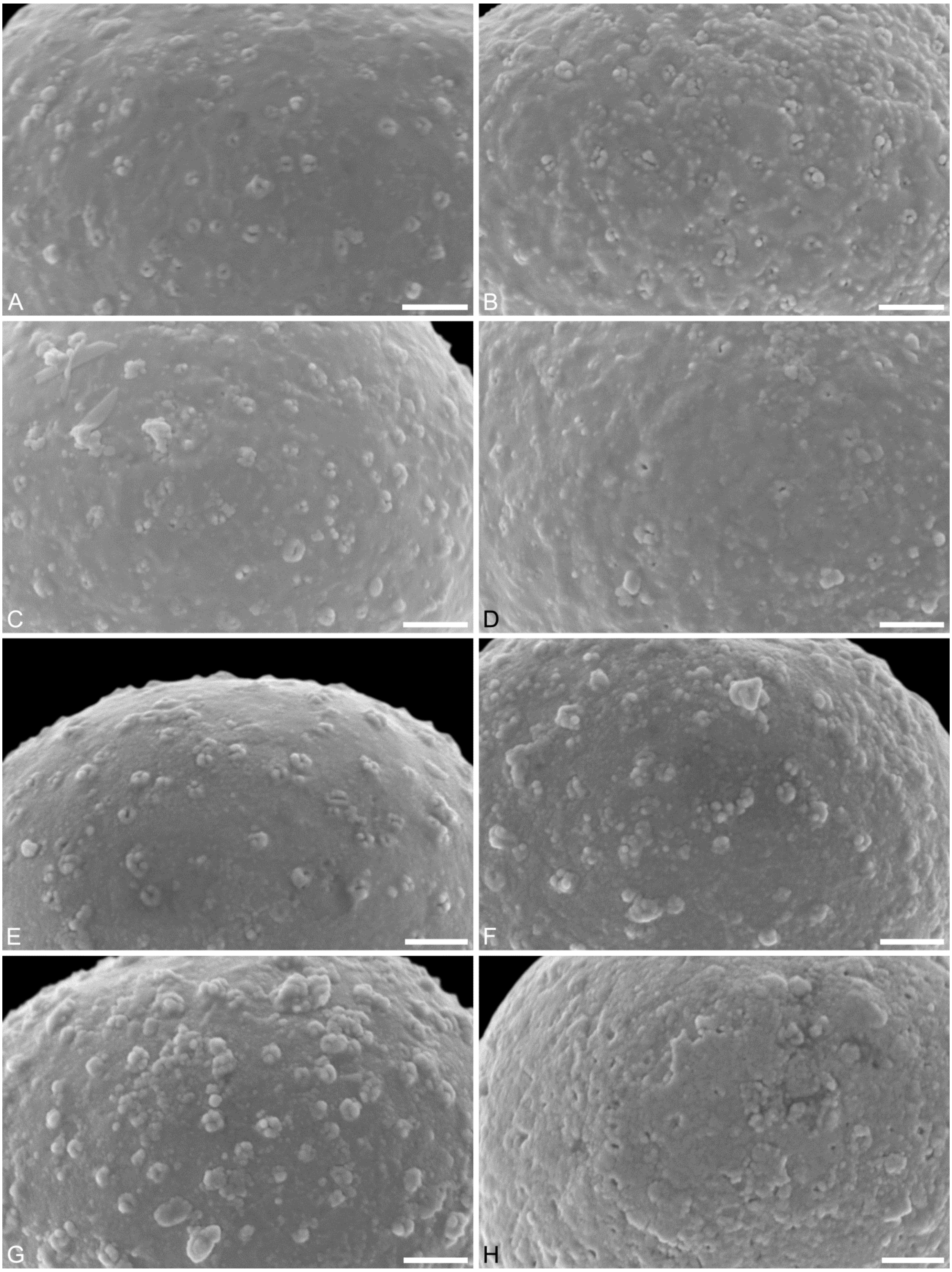

Figure S11. SEM micrographs of *Saururus cernuus* L. [WU 0039743]. IPUW 7513/129.

A–H) Close-ups of sculpture on proximal side of pollen.

Scale bars: 1 µm.

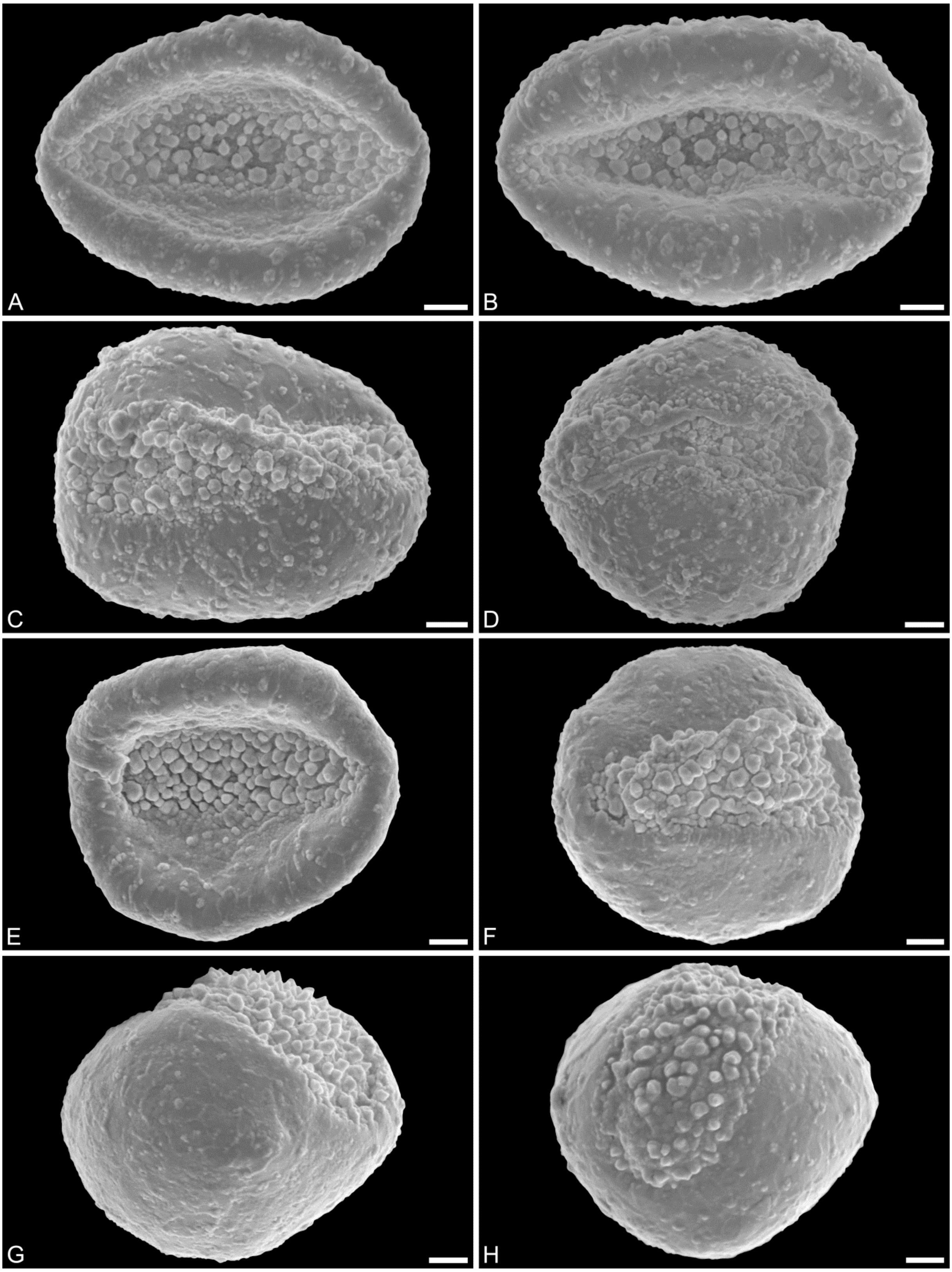

Figure S12. SEM micrographs of *Saururus chinensis* (Lour.) Baill. [WU 0039752]. IPUW 7513/130.

A–F) Pollen in distal polar view, showing sulcus and sulcus membrane.

G–H) Pollen in equatorial view.

Scale bars: 1 µm.

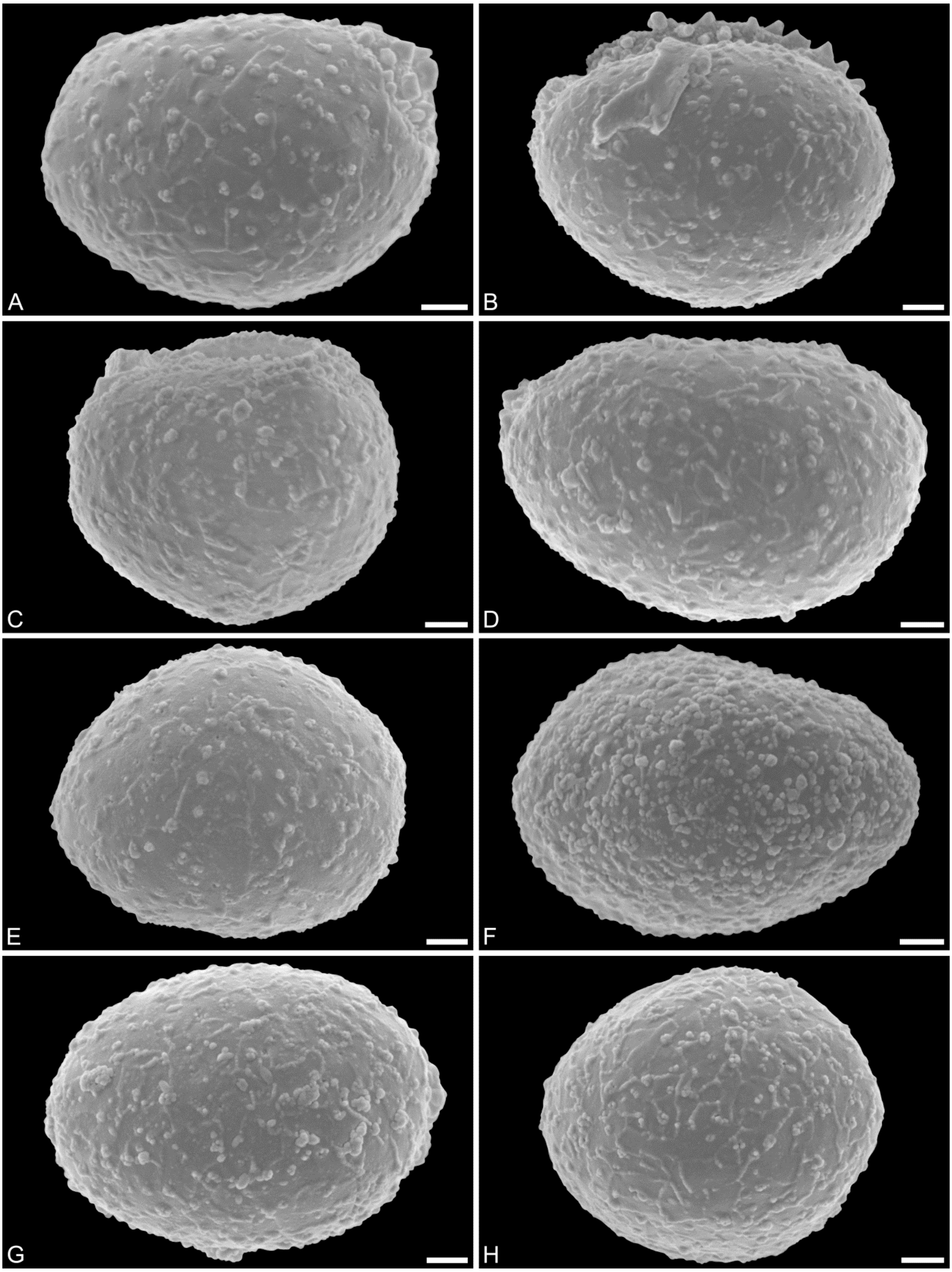

Figure S13. SEM micrographs of *Saururus chinensis* (Lour.) Baill. [WU 0039752]. IPUW 7513/130.

A–C) Pollen in equatorial view.

D–H) Pollen in proximal polar view.

Scale bars: 1 µm.

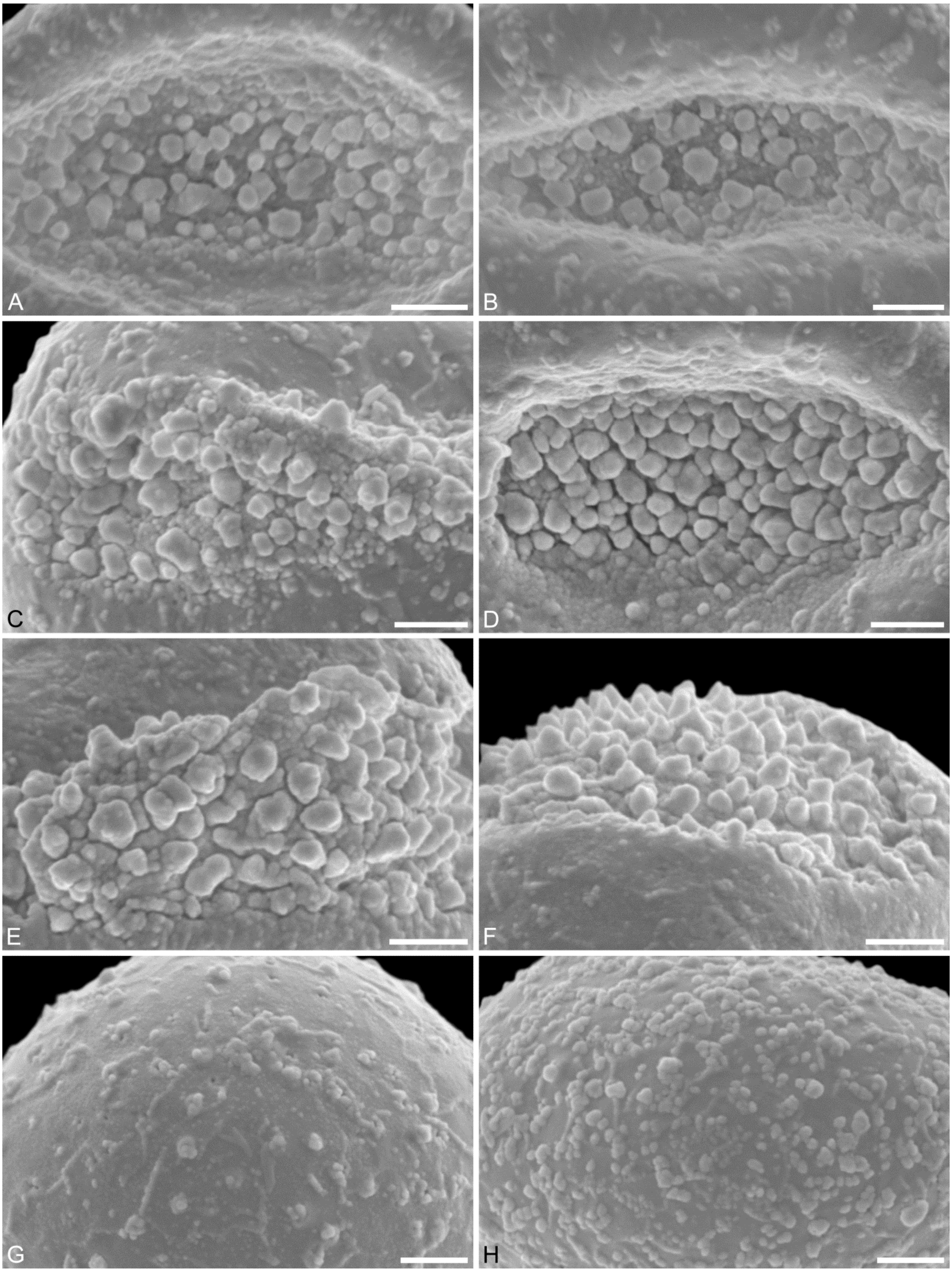

Figure S14. SEM micrographs of *Saururus chinensis* (Lour.) Baill. [WU 0039752]. IPUW 7513/130.

A–F) Close-ups of sulcus membrane.

G–H) Close-ups of sculpture on proximal side of pollen.

Scale bars: 1 µm.

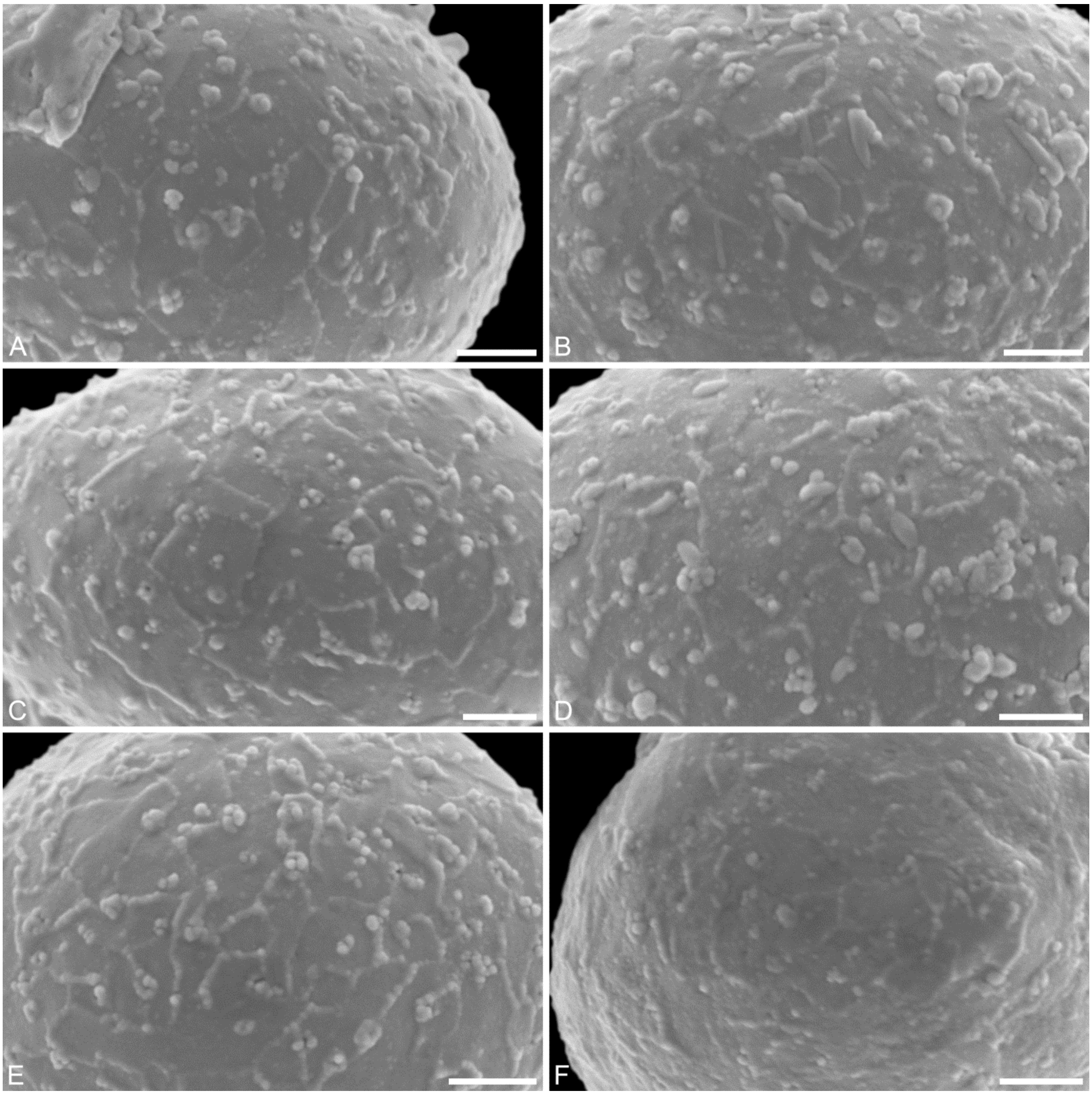

Figure S15. SEM micrographs of *Saururus chinensis* (Lour.) Baill. [WU 0039752]. IPUW 7513/130.

A–F) Close-ups of sculpture on proximal side of pollen.

Scale bars: 1 µm.
